# Adapting prime editing with split prime editors in *Escherichia coli* and its application to *Staphylococcus aureus* genome editing

**DOI:** 10.1007/s00253-026-13897-9

**Published:** 2026-06-04

**Authors:** Seong Hyeok Ma, Goosang Yu, Suyeon Park, Hyuna Sung, Uk Jin Jeong, Hyongbum Henry Kim, Junho Cho, Sang Sun Yoon

**Affiliations:** 1 Department of Microbiology and Immunology, Yonsei University College of Medicine, Seoul, 03722 Republic of Korea; 2 Department of Pharmacology, Yonsei University College of Medicine, Seoul, 03722 Republic of Korea; 3 Graduate School of Medical Science, Brain Korea 21 Plus Project for Medical Sciences, Yonsei University College of Medicine, Seoul, 03722 Republic of Korea; 4 Institute of Immunology and Immunological Diseases, Yonsei University College of Medicine, Seoul, 03722 Republic of Korea; 5 BioMe Inc, Seoul, 02455 Republic of Korea

**Keywords:** Prime editing, *Staphylococcus aureus* PE2, Methicillin-resistant *Staphylococcus aureus*

## Abstract

Prime editing is a precise and rapid genome-editing technique that modifies short DNA sequences using tailored guide RNAs. To implement this technique in bacteria, we used Prime Editor 2 (PE2) with the DeepPrime gRNA design tool and assessed its gene-editing efficiency in *Escherichia coli* and methicillin-resistant *Staphylococcus aureus* (MRSA) cells. Our findings indicate that a split PE2, comprising a reverse transcriptase and two Cas9 nickase domains, exhibited gene-editing efficiency comparable to that of the intact PE2. The efficiency observed in *E. coli* was significantly affected by the target sites, edit type, and the presence of exonucleases. In MRSA, which serves as a model to evaluate the applicability in non-model bacterial species, Streptococcus pyogenes PE2 (SpPE2) exhibited superior performance relative to *Staphylococcus aureus* PE2 (SaPE2). Furthermore, the split SpPE2 lacking the reverse transcriptase successfully induced the intended mutation in MRSA. This study demonstrates the feasibility of prime editing within bacterial systems.

**Supplementary Information:**

The online version contains supplementary material available at 10.1007/s00253-026-13897-9.

## Introduction

CRISPR-Cas9-based genome editing has been widely adopted across diverse fields, including metabolic engineering, high-throughput screening methods, and cell therapy, due to its effectiveness and rapid gene manipulation capabilities. Nevertheless, CRISPR-Cas9 systems encounter significant limitations attributable to high off-target effects and low homology-directed repair (HDR) efficiency (Wang and Doudna 2023).

Recently, prime editing (PE) has been developed to surpass these limitations. This system performs all types of editing—substitutions, deletions, and insertions—through its unique mechanism. It introduces a nick in a single DNA strand adjacent to the protospacer adjacent motif (PAM) sequence and interacts with the target DNA via base-pairing, mediated by the PE guide RNA (pegRNA). This capability enables precise genomic modifications without the need for double-strand breaks or donor DNA templates. PE2, the most widely used Prime Editor, utilizes a *Streptococcus pyogenes* Cas9 (SpCas9) nickase fused with a *Moloney murine leukemia* virus reverse transcriptase (M-MLV RT) that contains five distinct point mutations (D200N, T306K, W313F, T330P, and L603W). While numerous versions of PE have been developed, PE2 has been extensively validated not only in mammalian cells but also in various systems such as bacteria, yeast, and plants (Anzalone et al. 2019; Huang and Liu 2023).

In eukaryotes, the initial challenges of low efficiency and the complexity involved in the design of pegRNA for PE have been effectively reduced (Kim et al. 2021; Mathis et al. 2023). These advancements have facilitated the initiation of clinical trials employing PE techniques for the treatment of monogenic disorders, including chronic granulomatous disease (Gori et al. 2026). However, therapeutic applications face delivery challenges arising from the large size of PE systems. To address this, split PE systems alongside smaller Cas and RT proteins have been utilized (Lan et al. 2023).

In contrast to the progress observed in eukaryotic systems, PE has not been extensively investigated in prokaryotic systems due to its lower utility and efficiency compared to established methodologies, such as homologous recombination and CRISPR-Cas9 (Jiang et al. 2015). Recent studies have demonstrated that the deficiency of 3′ → 5′ exonucleases can enhance the efficiency of PE in bacterial systems, mirroring findings reported in mammalian cells (Zhang et al. 2024). This suggests that PE possesses considerable potential for broader applications within bacterial contexts.

Here, we demonstrate the successful implementation of various PE systems, previously utilized in eukaryotes, in bacteria. We employed DeepPrime, a deep learning model originally developed to predict pegRNA efficiency in eukaryotic systems, (Yu et al. 2023b) to design effective bacterial pegRNAs. We also validated our approach through both in vitro and in vivo experiments, performing a cross-platform assessment of the applicability of this prediction tool. Furthermore, we used multiple strategies to address size-related limitations while maintaining PE efficiency, providing practical solutions to overcome protein delivery challenges in bacteria.

Finally, we successfully applied both conventional and split PE systems to *Staphylococcus aureus*, a species previously unexplored for PE applications. This expands the potential of PE beyond simple model organisms to clinically relevant bacterial species that have traditionally relied on homologous recombination for genome engineering.

## Results

### DeepPrime enables the design of pegRNAs for bacterial prime editing

The design of pegRNA is a crucial factor in determining prime editing efficiency. Since no bacterial pegRNA prediction tool exists, we selected a deep learning model called DeepPrime, trained on the largest mammalian cell dataset (Yu et al. 2023a). To improve pegRNA effectiveness, we decided to use an engineered PE guide RNA (epegRNA) (Nelson et al. 2022), which features a 3′ structural motif—a prequeuosine_1-1_ riboswitch aptamer (tevopreQ1)—to prevent degradation of the 3′ end of the pegRNA (Roth et al. 2007).

**Fig. 1:**
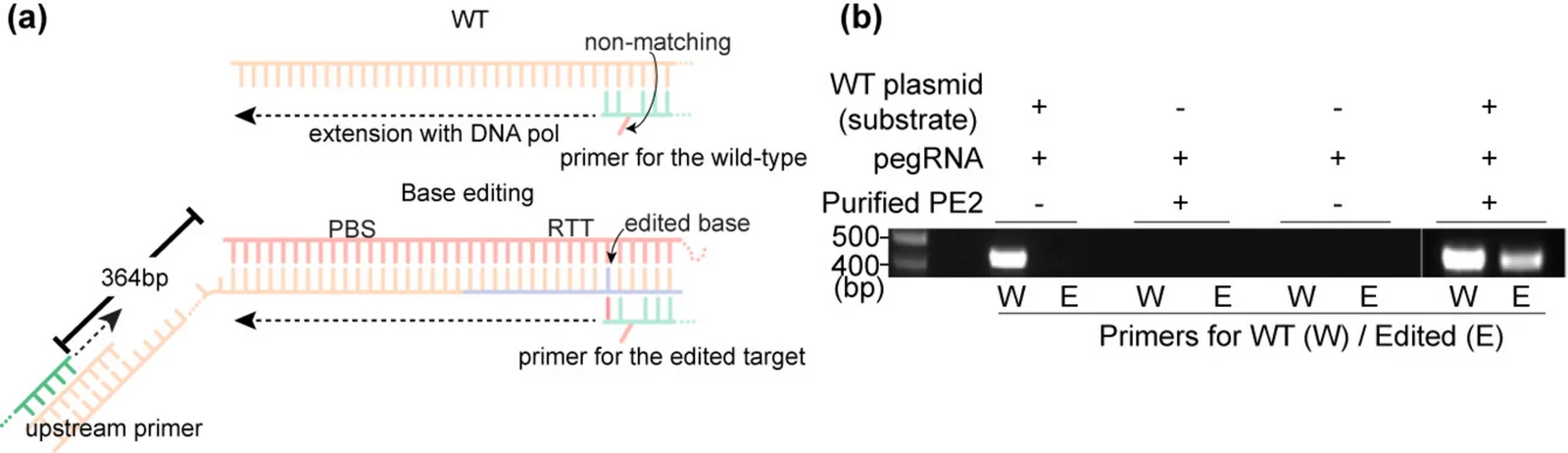
PegRNA design and in vitro prime editing assay. **a **Schematic representation of the in vitro prime editing assay. Two primers were designed to target both the wild-type (WT) and the edited sequences created through substitution. To enhance primer specificity, the −3 position of each PCR primer was modified to introduce a deliberate mismatch. To ensure accurate detection, the upstream primer is located 364 bp upstream of the epegRNA target site, yielding an expected PCR product of 436 bp. **b** Results of the in vitro prime editing assay. Lanes 1–6 represent negative controls (Lanes 1 and 2, lacking PE2; Lanes 3 and 4, lacking substrate; Lanes 5 and 6, lacking both PE2 and substrate). Successful amplification with the edit-specific primer occurred only when all components were present

To validate the DeepPrime-designed epegRNAs in vitro, we prepared purified PE2 (Supplementary Fig. 1a) and a target plasmid, pVIK112, where a G to T substitution is expected to occur at position + 1393 within the *lacZ* gene. For single-nucleotide polymorphism (SNP) detection, a customized PCR assay was used to distinguish mutant sequences carrying point mutations from the wild-type (Fig. 1a). We successfully obtained the desired PCR products by leveraging the specificity of the terminal base of the 5′ primers, along with modified PCR procedures. As the amount of the plasmid containing the substitution mutation increased, the amplification of PCR products became more significant when using the specific primer for that mutation (Supplementary Fig. 1b). We observed a clear PCR band only under conditions where purified PE2, epegRNA, and the target plasmid were all simultaneously present in the reaction, suggesting that all three components are essential for prime editing (Fig. 1b). These results indicate that the epegRNA, as designed with DeepPrime, can effectively induce prime editing in vitro, with the resultant editing being detectable in a simple and rapid manner, even with a 1-bp substitution.

### PE2 functions effectively in *E. coli* and MRSA

**Fig. 2:**
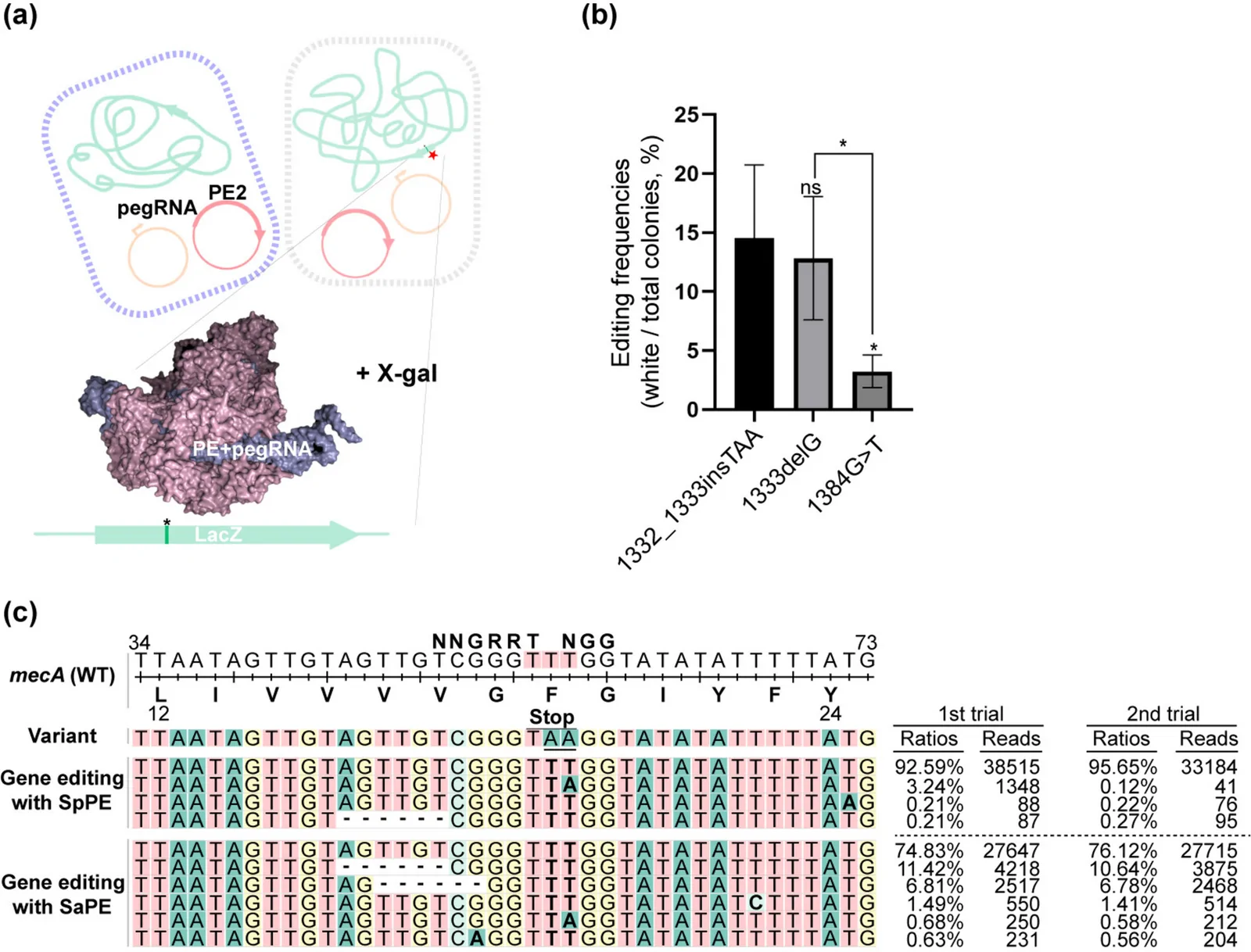
Validation of the prime editing system in *E. coli* MG1655 and MRSA JE2. **a** An illustration of blue-white screening used for validating the designed epegRNAs in *E. coli MG1655*. **b** Prime editing results utilizing epegRNAs designed by DeepPrime. Editing efficiency was measured by the ratio of white colonies to the total number of colonies. Data represent the mean ± standard deviation from *n* = 3 independent replicates. **c** Results of deep amplicon sequencing at the *mecA* mutant target site. The frequency of the intended edit is highlighted in gray. PAM sequences are shown as NNGRRT for SaPE2 and NGG for SpPE2

We investigated the efficiency of prime editing using epegRNAs in *E. coli*. To facilitate in vivo assays, two plasmids were constructed: one containing the epegRNA targeting the *lacZ* gene and the other encoding PE2 under the control of a tetracycline-inducible promoter. The intended editing introduces a premature stop codon by directly adding a TAA codon (1332_1333insTAA), inducing a frameshift via a single-nucleotide deletion (1333delG), or making a nonsense mutation through a G-to-A substitution at 1384 (1384G > T) (Supplementary Fig. 2a), resulting in a truncated β-galactosidase that cannot cleave X-gal as a substrate. Consequently, we measured efficiency by quantifying the number of blue and white colonies (Fig. 2a and Supplementary Fig. 2b). Our data showed variations in efficiency by edit type, with high rates for both deletion and insertion and a relatively low rate for substitution at this specific locus within the *lacZ* target (Fig. 2b). However, it should be noted that these editing efficiencies are highly context-dependent and can vary significantly depending on both the target gene and the specific loci being edited.

We further explored the application of prime editing to generate genetic variants in *Staphylococcus aureus*, a species where this technique is still underexplored. We chose the methicillin-resistant *S. aureus* strain JE2, a USA300 derivative, in which CRISPR/Cas9 editing is feasible (Chen et al. 2017).

To assist with selection, we initially generated a *mecA* mutation in JE2 by introducing a premature stop codon via homologous recombination, which resulted in the loss of the distinctive methicillin resistance characteristic of MRSA (Wielders et al. 2002). Subsequently, we built a prime editing system that expresses the SpPE2 protein controlled by an anhydrotetracycline (ATc)-inducible promoter, along with a cognate epegRNA driven by the *rrnB* promoter. The epegRNA, selected for the highest DeepPrime score (Supplementary Table S1), was designed to revert the *mecA* mutation to the wild-type sequence. Successful editing restored antibiotic resistance, allowing selection on a plate containing ampicillin (Amp). Due to the relatively low efficiency of prime editing in MRSA compared to *E. coli*, directly detecting edited cells on non-selective medium was challenging. Thus, we enriched the edited population by culturing the cells overnight and plating them on an ampicillin-containing rich medium. Notably, no colonies were observed on the ampicillin plates in the negative control lacking epegRNA, confirming that the surviving colonies were the result of prime editing (Supplementary Fig. 3). We determined the outcome of prime editing using deep amplicon sequencing and achieved the desired reverted mutation with high specificity of up to 95.65% at the target sites (Fig. 2c). We also examined the editing performance of a prime editor based on SaCas9, which originates from *S. aureus* (SaPE2), resulting in reduced editing efficiency with a specificity of only 76.12%.

Additionally, we evaluated the editing performance of SpPE2 and SaPE2 in *E. coli* and found their overall efficiencies to be comparable (Fig. 2b and Supplementary Fig. 4). However, it should be noted that a direct comparison between the two systems is limited. Because SpCas9 and SaCas9 recognize different PAM sequences (NGG and NNGRRT, respectively) and thus require distinct epegRNA designs, an absolute comparison is challenging. Collectively, our results indicate that epegRNAs, designed using a gRNA prediction model developed for mammalian cells, can be effectively applied with SpPE2 in bacterial systems.

### PE2 with RT variants operates similarly to intact PE2

Given its precision, prime editing has been regarded as a promising approach for gene therapy. However, the large size of the prime editor presented an obstacle to AAV packaging. (Liu et al. 2022) Recent research has focused on shrinking the editor’s size without sacrificing its editing efficiency. (Gao et al. 2022; Lan et al. 2023).

Prior to evaluating the split prime editing system in bacteria, we first constructed a 3′ to 5′ exonuclease-deficient strain *(E. coli MG1655* Δ*sbcB*, Δ*exoX*, Δ*xseA*), previously validated to improve prime editing efficiency (Zhang et al. 2024). Interestingly, when we tested the epegRNA that showed the highest editing efficiency in Fig. 2b, the single knockout of *sbcB* unexpectedly resulted in decreased efficiency at this specific *lacZ* target site. While this single-knockout effect may be a site-specific phenomenon, knocking out all three exonucleases (*sbcB*, *exoX*, *xseA*) resulted in a substantial improvement in overall editing efficiency, increasing it by 5.97-fold (Supplementary Fig. 5a).

We additionally recognized that blue-white screening has a limitation, which leads to mixed colonies containing both non-edited and edited cells on the agar plates. Correspondingly, we incorporated a frame-shifted kanamycin resistance gene (*KanR*) into a phenotypically neutral target site, the *ybcC* locus, using CRISPR-λ Red engineering. This served as another editing selection marker, similar to the *mecA* mutation selection in JE2. Editing efficiency was evaluated by the proportion of colonies surviving on Kan + plates compared to the total number on Kan − plates (Supplementary Fig. 5b). Using these two strategies, we targeted both the *lacZ* and *kan*^*R*^Δ66–67 to assess editing efficiency.

**Fig. 3:**
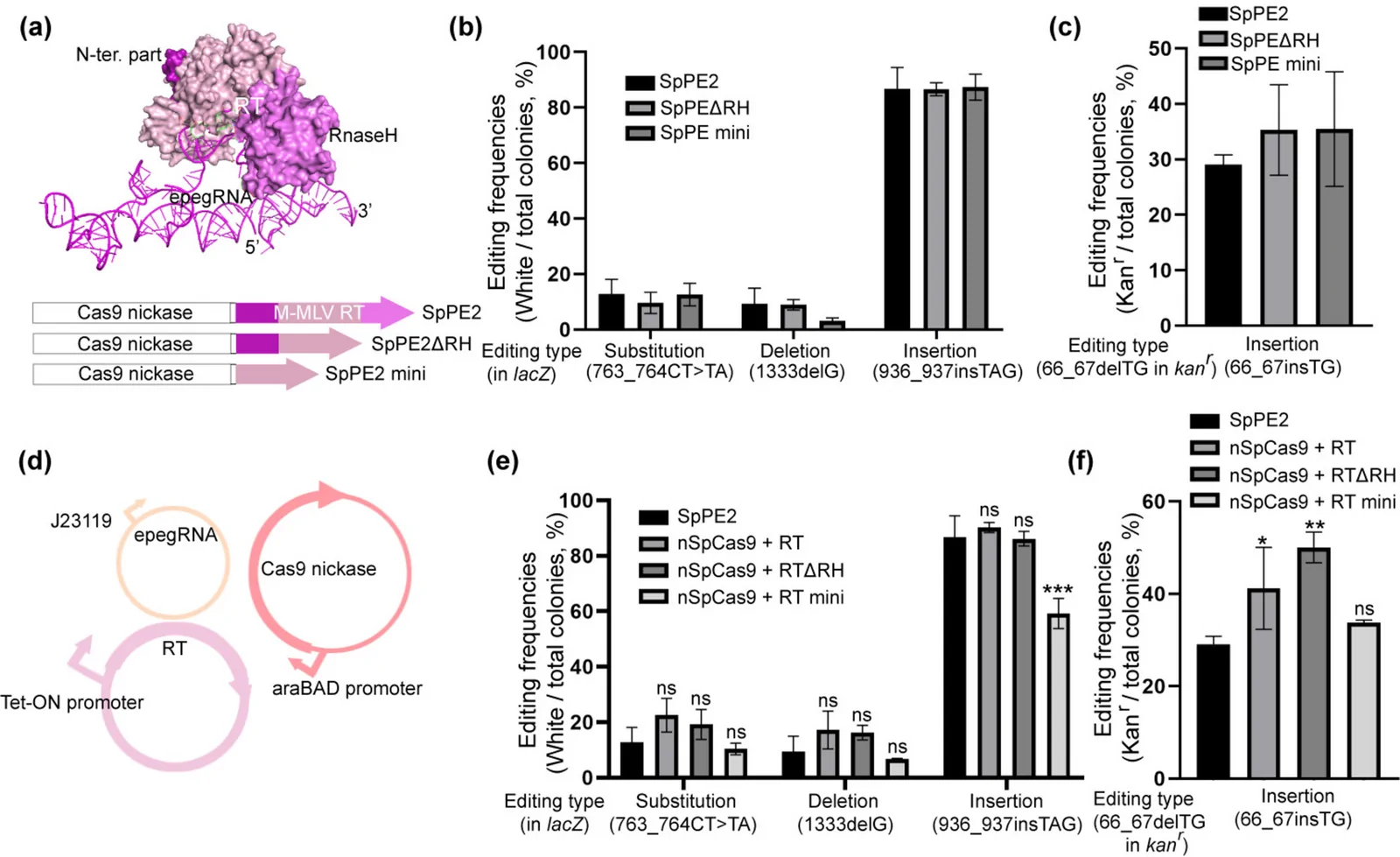
Prime editing with SpPE2-RT variants and untethered PEs. **a** An illustration of SpPE2 variants. Among the M-MLV RT regions, the removed RNase H and N-terminal domains are shown in pink. The SpPEΔRH was a SpPE2 variant devoid of the RNaseH domain, and the SpPE mini additionally lacked the N-terminal region. **b**, **c** Prime editing efficiencies targeted at the *lacZ* (b) and *kanR* (c) loci. Data represent mean ± s.d. of *n* = 3 independent replicates. **d** Plasmids for expression of untethered PEs. The epegRNA, Cas9 nickase, and M-MLV RT variants were expressed under the control of the J23119, arabinose-inducible, and tetracycline-inducible promoters, respectively. **e**, **f** Prime editing with untethered PEs targeting *lacZ* (**e**) and *kanR* mutant (**f**). Data represent mean ± s.d. of *n* = 3 independent replicates

SpPE2 consists of an SpCas9 nickase (nSpCas9) and an engineered M-MLV reverse transcriptase (RT). The M-MLV RT contains an RNase H domain, and removing this domain does not affect the RT’s functionality for prime editing (Gao et al. 2022; Grünewald et al. 2023). We investigated two variants of PE2 with truncated RTs that had previously shown activity in human cells: SpPE2 without the RNase H domain (SpPE2ΔRH) and SpPE2ΔRH lacking the N-terminal region of the RT (SpPE mini) (Fig. 3a). Both SpPE2ΔRH and SpPE mini exhibited editing efficiencies comparable to SpPE2 at the *lacZ* locus, except for the deletion mutation, where SpPE mini showed a reduced trend (Fig. 3b). Under antibiotic selection with kanamycin, the insertion editing efficiencies of both variants were around 35%, comparable to those of SpPE2 (Fig. 3c).

Furthermore, we assessed whether a split PE system, which involves removing the linker between nSpCas9 and MMV-RT, could also maintain its prime editing capability in a bacterial system. To examine this, we constructed three plasmids that express the SpCas9 nickase under an arabinose-inducible promoter, the untethered RT under an ATc-inducible promoter, and epegRNAs under a J23119 promoter, respectively (Fig. 3d).

Before assessing the split PE system, we established the optimal conditions for expressing each protein. In the absence of the two inducers (arabinose and ATc), editing appeared to be inhibited, showing approximately 2% editing efficiency (Supplementary Fig. 5c). We determined that final concentrations of 10 mM arabinose and 100 ng/µl ATc were optimal, based on our observation that editing efficiency reached up to 73.26%. In combination with the RT variants, we observed an overall increase in editing efficiency, except for SpPE mini, compared to the conventional PE system, with the highest efficiency seen when combined with RTΔRH (Fig. 3e and f).

### Split PE2 and its variants retain their gene-editing ability in *E. coli* and MRSA

**Fig. 4:**
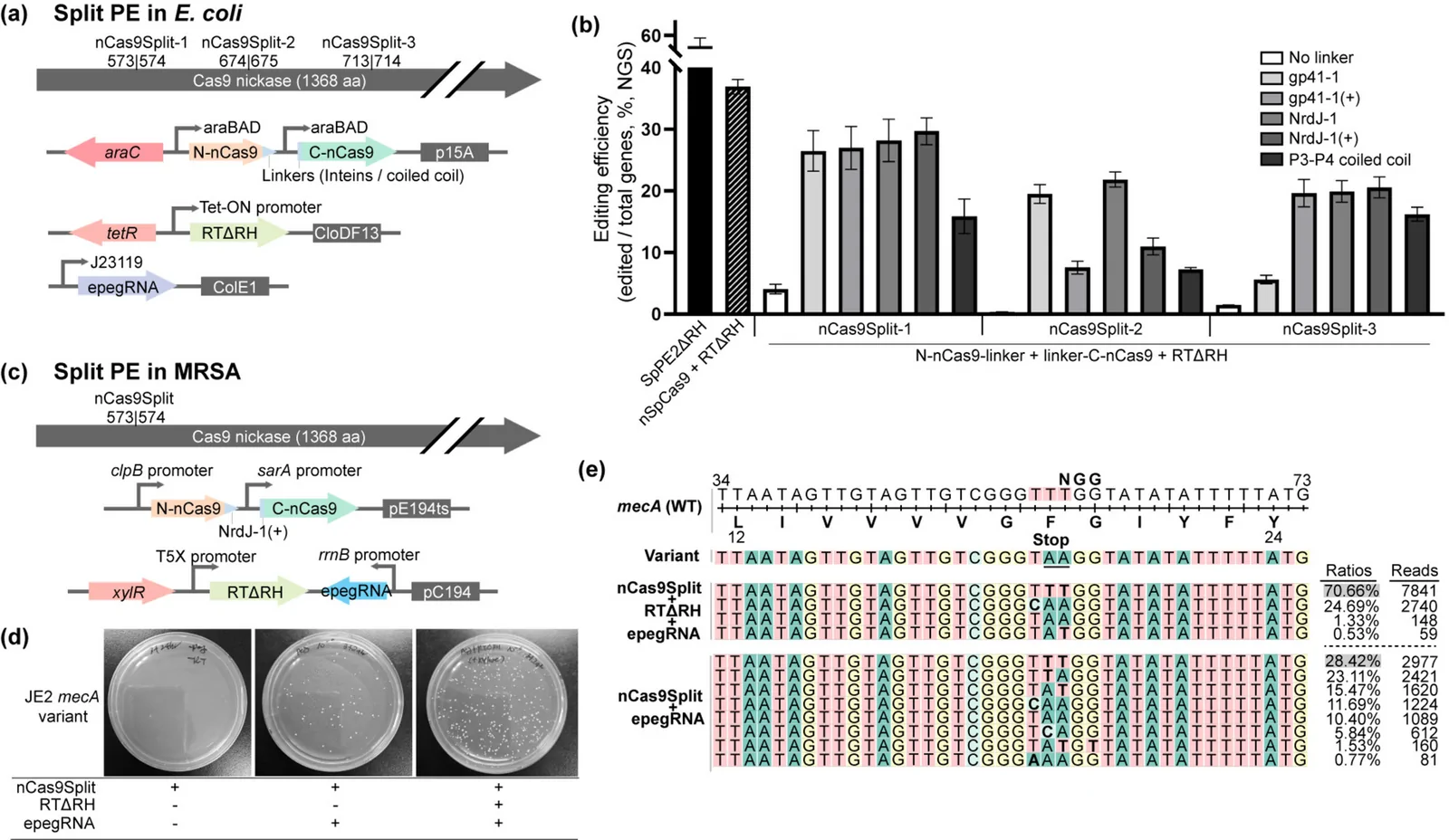
Validation of the split prime editing system in *E. coli* MG1655 and MRSA JE2. **a** Schematic of the split PE system in *E. coli* MG1655. **b **Prime editing with the split PE system targeting the kanR mutant. Inteins (gp41-1 and NrdJ-1) or coiled-coil domain (P3-P4) were added to increase the activity of split nCas9. The inclusion of native intein junction sequences is indicated by (+). Data represent mean ± s.d. of *n* = 3 independent replicates. **c** Schematic of the split PE system in MRSA JE2. **d** Representative colonies on the amp (+) plates according to the different prime editing components. **e** Result of deep amplicon sequencing at the mecA mutant target site using the split PE system. The frequency of the intended edit is highlighted in gray

To the best of our knowledge, the functionality of PE2, when its components are expressed separately as the N-terminal and C-terminal halves of the Cas9 nickase, alongside the reverse transcriptase (Fig. 4a), has not yet been confirmed in bacterial systems. We aimed to determine whether the separated domains also retain their activity.

We employed three previously established split sites within nSpCas9, where enzymatic activity could be maintained by using adapters such as inteins and heterodimers in human cells. (She et al. 2023; Truong et al. 2015; Yuan et al. 2022; Zetsche et al. 2015) Previous studies identified an intein library that showed splicing activity in *E. coli.* (Pinto et al. 2020) From this library, we selected two ultrafast inteins, gp41-1 and NrdJ-1, which were discovered through metagenomic data and show more rapid and efficient protein splicing than the inteins natively found in bacteria. Additionally, we adopted a P3-P4 heterodimer, which was previously used to connect nSpCas9 and M-MLV-RT (Mu et al. 2024), though it had not been applied to split Cas9 (Fig. 4a). With these adapter-mediated split nSpCas9s, we integrated RTΔRH, attaining maximum efficiency in the split PE system (Fig. 3e and f).

To accurately quantify editing efficiencies, next-generation sequencing (NGS) was performed on all colonies transformed with the plasmids. In the absence of adapters, we observed minimal editing of the *lacZ* gene, with only 4.07% of the intended edits occurring at the nCas9Split-1 site (573N/574C) in *E. coli* (Fig. 4b). The prime editors that incorporated adapter-mediated Cas9 showed significantly enhanced editing efficiencies, achieving nearly 30% at the nCas9Split-1 site and 20% at other sites. However, these results did not surpass those of SpPE2ΔRH or the non-split nSpCas9 with RTΔRH.

To account for the influence of flanking residues on intein activity, we incorporated a native three-amino-acid junction sequence. (Carvajal-Vallejos et al. 2012) Subsequent evaluation revealed that the effect of these junction sequences on their splicing and editing efficiency varied significantly depending on the split site (Fig. 4b, nCas9Split-2 and -3). We then attempted to introduce this system into MRSA by expressing N-Cas9 and C-Cas9 under the control of strong constitutive promoters, *clpB* and *sarA*, respectively. Additionally, RTΔRH was expressed under a xylose-inducible promoter (Fig. 4c). Our findings showed that the split PE2 system achieved the intended edits at a ratio of 70.66%. However, when RTΔRH was absent, the rate dropped to 28.42%. Without epegRNA, we did not observe any intended mutations (Fig. 4d and e). These results indicate that prime editing can occur in MRSA, potentially through a distinct mechanism from that in *E. coli*, such as differences in the mismatch repair system. In conclusion, we demonstrated that the split PE system is functional and maintains its efficiency not only in *E. coli* but also in MRSA.

## Discussion

We present a comprehensive overview of advanced CRISPR-Cas9 technology, specifically prime editing, for bacterial genome editing applications. While we utilized DeepPrime (Yu et al. 2023b)—a predictive model highly effective for human cell editing—to design our pegRNAs, we acknowledge that its predictive accuracy and exact correlation with actual editing efficiencies in bacterial systems has yet to be comprehensively established due to species-specific DNA repair mechanisms and distinct genomic compositions. Nevertheless, we hypothesize that this model would maintain robust performance, given that prime editing has been effectively demonstrated in *Escherichia coli*, *Klebsiella pneumoniae*, and *Acinetobacter baumannii* (Tong et al. 2021; Zhang et al. 2024).

To validate epegRNAs designed by DeepPrime, we developed an in vitro prime editing assay utilizing a SNP-specific PCR detection method (Chen and Schedl 2021). Unlike current approaches that frequently rely on fluorescent dyes (e.g., Cy5), our system employs non-compatible primers targeting wild-type and mutant, respectively, enabling cost-effective and rapid detection (Fig. 1c). Specific amplicons were generated exclusively when reactions were supplemented with purified PE2–epegRNA ribonucleoprotein (RNP) complexes (Fig. 1d). Furthermore, we validated the system’s functionality using a split prime editor: Upon reconstituting nSpCas9 split at residues 535N/536C via a GCN4 homodimer, successful editing was confirmed by PCR detection (Supplementary Fig. 6).

In light of our successful in vitro and in vivo experiments, we explored a plasmid-free prime editing technique using purified PE2 and epegRNAs. Genome editing with ribonucleoproteins (RNPs) has demonstrated effective gene-editing capabilities in human cells, reducing off-target effects (Ponnienselvan et al. 2023). We hypothesize that protein delivery into bacteria via electroporation would be feasible and could ultimately result in gene editing, as proteins are fundamentally smaller than DNA. However, efforts to implement this technique in bacterial cells have not met with success. Given that editing was achieved when the prime editor was expressed in *E. coli* and epegRNA was subsequently delivered, we reason that this outcome may be attributed to the smaller size of bacterial cells and their distinct, extensive membrane architecture compared to mammalian cells (Supplementary Fig. 7). Nevertheless, previous studies have shown that T7 RNA polymerase (98 kDa) is still effectively introduced through electroporation into *E. coli* DH5α, while larger protein size negatively affects delivery efficiency (Crawford et al. 2013; Sustarsic et al. 2014). Considering that prime editors such as SpPE2 typically have a molecular weight of approximately 237 kDa, these findings support the idea that minimizing the size of PEs may enhance their delivery and functionality in bacterial systems.

We applied several strategies previously validated in human cells to optimize the size and modularity of SpPE2 for use in bacterial cells. First, we engineered a truncated version of M-MLV RT (Fig. 3a) and removed the linker between nSpCas9 and MMLV-RT (Fig. 3d). Additionally, we adopted a split-Cas9 approach (Fig. 4a), which allowed further size reduction. In line with previous studies, we tested three split sites (573N/574C, 674N/675C, 713N/714C) known to be effective for Cas9 nickase reconstitution. Since the enzymatic activity of split Cas9 drops significantly without adapters, we utilized inteins (gp41-1 and NrdJ-1) and heterodimerization domains (P3 and P4) to facilitate the reassembly of split Cas9 and restore its editing function.

We validated two truncated versions of SpPE2: SpPE2ΔRH, which lacks the RNase H domain, and SpPE2 mini, which is further truncated at the N-terminal region of M-MLV RT. Both versions maintained prime editing efficiencies similar to the full-length SpPE2 (Fig. 3b and c). Notably, we observed discrepancies between the results from kanamycin selection and deep sequencing for SpPE2ΔRH and the untethered PE with RTΔRH (Figs. 3c and 4b). This might be due to fundamental differences between the measurement methods. Unlike kanamycin selection, which imposes irreversible pressure during plating and immediately determines cell survival based on editing status, deep sequencing requires additional time during plating, which could increase the chance of editing occurring before DNA extraction and analysis.

When split Cas9 was used for prime editing, we found certain variants that maintained relatively high efficiencies of around 30%, despite overall reductions in editing performance (Fig. 4a and b). Incorporating the native junction sequence of the intein led to either an increase or a decrease in efficiency, depending on the split site. Since the added sequence remains after protein splicing, it is likely to have influenced the prime editor’s structural function.

Finally, we applied the PE systems reconstituted in *E. coli* to MRSA, a bacterium where prime editing has not been previously used. With SpPE2, we observed successful editing in the MRSA JE2 strain (Fig. 2c). Since SaCas9, derived from *S. aureus*, is known for its higher inherent editing efficiency, we developed a prime editor based on SaCas9 (SaPE2) and compared its efficiency and specificity to SpPE2. Surprisingly, SaPE2 showed lower specificity than SpPE2. We also validated the split SpCas9 variant 573N/574C_NrdJ-1(+), which had the highest editing efficiency in *E. coli*, showing a similar pattern, though with reduced specificity (Fig. 4e). Unexpectedly, colonies appeared when attempting prime editing without RTΔRH (Fig. 4d). While the precise mechanism remains to be fully elucidated, we propose three plausible mechanisms for this RT-independent editing in MRSA: (1) the presence of uncharacterized endogenous reverse transcriptases, such as group II introns that can utilize the epegRNA template; (2) endogenous nick-repair pathways; or (3) atypical DNA repair processes driven by native polymerases. Our findings suggest that prime editing employs distinct mechanisms not only across different editing systems but also between species. In mismatch repair, exonucleases closely associated with prime editing differ between species. In *E. coli*, multiple exonucleases such as *ExoI*, *RecJ*, and *ExoX* are involved in the repair process, whereas in *S. aureus*, only RecJ is present (Ha and Edwards 2021). In addition, unlike *E. coli*, *S. aureus* is likely to utilize a MutH-independent repair pathway and the presence of an additional main polymerase, PolC, which is involved in the repair system. This unique repair machinery strongly supports our third hypothesis, as PolC might atypically synthesize the edited strand by directly tracking the epegRNA. Furthermore, the *S. aureus* genome is composed of nearly 70% AT content (Hurley et al. 2023), which suggests that further study is necessary to elucidate the impact of an AT-rich genome on prime editing outcomes. These fundamental differences support the notion that *S. aureus* exhibits distinct patterns from *E. coli*, as discussed above.

This study focused on the applications of prime editors to bacteria and the evaluation of various prime editor architectures rather than the comprehensive validation of machine-learning prediction models in bacteria. Our successful use of the epegRNAs for proof-of-concept editing suggests the potential utility of DeepPrime as a preliminary design tool for bacterial systems. Further large-scale correlation analyses will be required in future studies to evaluate and adapt these models for prokaryotes.

Most bacterial genome editing tools have been developed and tested in a few model organisms such as *E. coli*. Yet most commensal bacteria remain difficult to engineer because they lack plasmids for DNA delivery and selection. Therefore, a logical next step is to explore plasmid-free approaches in which the editing machinery is introduced as pre-assembled RNPs. Prime editing offers the required precision, and splitting the prime editor into smaller fragments should allow assembly of RNPs that are easier to make and deliver. We anticipate that such a modified PE RNP could introduce precise changes in non-model bacterial species without relying on a plasmid. This strategy would minimize horizontal transfer risk and avoid the need for strain-specific plasmid optimization. By outlining this concept, we aim to encourage further work that will broaden gene editing to a wider range of microbiome members and support future studies on strain improvement and functional analysis.

## Methods

### Bacterial strains, plasmids, primers, and culture conditions

The strains, primers, and plasmids used in this study are listed in the Supplementary Tables S2 and S3. All *E. coli* strains were cultured in Luria–Bertani (LB) broth (BD Difco #244620). *Staphylococcus aureus* JE2 was grown in Tryptic Soy Broth (TSB) (BD Difco #211825). The following concentrations of antibiotics and inducers were used: ampicillin (100 µg/ml), chloramphenicol (20 µg/ml), spectinomycin (50 µg/ml), kanamycin (50 µg/ml), anhydrotetracycline (100 or 200 µg/ml), and L-arabinose (10 mM). For blue-white screening, X-gal and IPTG were added to the agar plates at final concentrations of 100 µg/ml and 0.1 mM, respectively.

### Plasmid construction

The base plasmid used for PE was pCRISPR-PE bacteria (Addgene #132730), which encodes the SpPE2 protein. To generate variants of SpPE2, we employed a site-directed mutagenesis kit (Enzynomics #EZ004S) to delete the RNase H domain, resulting in the SpPE2ΔRH variant. For constructing the mini version of SpPE2, we used Gibson assembly to truncate non-essential regions of the N-terminal part from M-MLV RT.

To create split prime editor (split PE) systems, the linker and reverse transcriptase (RT) regions were removed from pCRISPR-PE bacteria through site-directed mutagenesis. We cloned nSpCas9 under the control of the arabinose-inducible araBAD promoter. The RT was cloned separately into a pCDF-based plasmid under the control of a tetracycline-inducible promoter, along with the TetR repressor.

For the split nSpCas9 system, each fragment of N- or C-nSpCas9 was first cloned into pBAD-based plasmids using Gibson assembly. The two fragments were then combined into a single plasmid. The pCRISPR-SaPE (human or Streptomyces codon) constructs were generated using nSaCas9 from pLenti-BSD and pCRISPomyces-SaCas9 (Addgene #129553). We assembled pegRNA plasmids using Gibson assembly for expression under the pJ23119 promoter, utilizing either the conventional or optimized scaffold.

### Strain construction

*E. coli* mutant strains were constructed using the pKD46-Cas9 system (Jiang et al. 2015). A spacer was cloned into pTargetF and then electroporated along with donor DNA into *E. coli* MG1655 that harbored the pCas plasmid. The electroporated cells were plated on LB agar containing 50 µg/ml of spectinomycin and 50 µg/ml of kanamycin, followed by colony PCR to pick colonies for verification of gene deletion. After confirming the deletion, the pCas and pTargetF plasmids were eliminated by culturing the mutants overnight without antibiotics. To check for plasmid curing, overnight cultures were plated on LB agar at appropriate dilutions and streaked for colonies, with one plate containing spectinomycin (50 µg/ml) and kanamycin (50 µg/ml) and another without antibiotics. To generate MRSA mutants, a thermosensitive pMAD vector for allelic replacement and the *E. coli* strain IM08B, used as a shuttle host, were employed following previous studies (Arnaud et al. 2004; Monk et al. 2016). In summary, approximately 1000 bp fragments flanking upstream and downstream of the target gene were amplified and subsequently inserted into the pMAD vector utilizing *BamHI* and *XmaI* restriction enzymes, followed by ligation with T4 DNA ligase. The JE2 strain was transformed using the newly constructed pMAD vector, followed by the homologous recombination procedures as reported in previous studies. The JE2 transformants were initially grown at 30 °C overnight. To promote chromosomal integration, the overnight culture was inoculated into 5 ml of TSB, incubated at 42 °C for 6 h, and then plated on agar medium containing 2.5 µg/ml erythromycin and 50 µg/ml X-gal, followed by another overnight incubation. Light blue colonies were selected, reinoculated, and grown in 5 ml of TSB at 30 °C to facilitate plasmid curing. The grown cells were then spread onto TSA plates containing X-gal without the selection marker. White colonies were restreaked onto two types of TSA plates (with and without erythromycin). Finally, the erythromycin-sensitive white colonies that only grew on the plates without erythromycin were selected as the final mutants and confirmed by DNA sequencing.

### epegRNA design

Target candidates containing PAM sequences were scored using DeepPrime (Yu et al. 2023b). Next, optimized linkers for tevopreQ1 attachment were identified with pegLIT (Nelson et al. 2022). The resulting epegRNAs were then analyzed for secondary structure formation using UNAfold (Markham and Zuker 2008).

### PE2-9His expression and purification

*E. coli* ArcticExpress RIL (DE3) containing the pET21b-PE 9His plasmid was pre-cultured in 10 mL of LB medium at 37 °C. A 2 mL aliquot of the pre-culture was inoculated into each of five 1-L flasks containing 200 mL of LB medium. The cells were grown at 37 °C until they reached an optical density at 600 nm (OD_600_) of approximately 0.4. At this point, protein expression was induced by adding IPTG to a final concentration of 0.4 mM. The cultures were incubated at 16 °C for 20 h. Following incubation, the cells were harvested by centrifugation at 8000 rpm for 5 min at 4 °C using a high-speed centrifuge. The resuspended cells were stored at −80 °C until needed.

The pellet was resuspended in 10 mL of lysis buffer, which contained 50 mM Tris–HCl (pH 7.5), 500 mM NaCl, 1 mM imidazole, 10 mM MgCl_2_, 1 mM PMSF, a 1 × protease inhibitor cocktail (Sigma), and 1 mg/mL of lysozyme. The mixture was incubated for 30 min at 4 °C. Cells were then lysed using a Branson sonicator (6 mm probe) with the following parameters: 10-s on and 10-s off pulses, an amplitude of 20–30%, for a total sonication time of approximately 10 min (either 4 min in 2 segments or 2 min in 5 segments).

The lysates were clarified by centrifugation at 13,000 rpm for 30 min at 4 °C. The supernatant was collected and mixed with Ni–NTA resin (Qiagen #30,210) that had been pre-equilibrated with the pre-lysis buffer (50 mM Tris–HCl, pH 7.5, 500 mM NaCl, and 1 mM imidazole). The resin and lysate mixture were incubated overnight at 4 °C with gentle shaking to enhance binding efficiency. The following day, the flow-through was collected, and the mixture was reapplied to the column to maximize binding.

Sequential washes were performed using a gradient imidazole wash buffer (50 mM Tris–HCl, pH 7.5, 500 mM NaCl) containing 10, 25, and 50 mM imidazole. Elution was carried out with an elution buffer (50 mM Tris–HCl, pH 7.5, 500 mM NaCl, 500 mM imidazole), and the elution fractions were analyzed by SDS-PAGE. Fractions that contained high yields of PE2-9His were concentrated and buffer-exchanged using a 30 kDa cut-off Vivaspin column (Sartorius). Finally, the concentrated proteins were stored in a 1 × storage buffer, which included 10 mM Tris–HCl (pH 8.0), 100 mM NaCl, 0.1 mM EDTA, 1 mM DTT, and 30% glycerol.

### epegRNA expression and purification

The epegRNAs were cloned under the T7 promoter using Gibson assembly to perform in vitro transcription. The PCR was conducted from the T7 promoter to the tevopreQ1 sequence, and the resulting PCR product was used in the reaction. In vitro transcription was carried out at 37 °C using the HiScribe T7 High Yield RNA Synthesis Kit (New England Biolabs #2040S). The reaction was then purified using the MEGAclear Transcription Clean-Up Kit (Thermo Scientific #AM1908).

### In vitro prime editing assay

In vitro prime editing was conducted using the pVIK112 plasmid as the substrate, targeting a G to T substitution at position + 1393 within the lacZ gene. Before the reaction, the epegRNA and prime editor proteins were pre-incubated in NEBuffer 3.1 (New England Biolabs) at 25 °C for 15 min. After this, the plasmid substrate and dNTPs were added to the mixture, followed by incubation at 37 °C for 1 h to facilitate the editing reaction.

The resulting products from this reaction were subjected to PCR amplification using Dyne Taq polymerase (Dyne Bio, DYP1020) and site-specific primers. The PCR was conducted under the following conditions: an annealing temperature of 60 °C, an elongation time of 30 s, and a total of 20 amplification cycles. To improve primer specificity for the edited sequence, intentional mismatches were introduced at the −3 position of each primer (Chen and Schedl 2021). Finally, the PCR products were analyzed by agarose gel electrophoresis to confirm the presence of the desired editing outcome.

### Prime editing

For prime editing in *E. coli*, cells were transformed with a plasmid that expresses the prime editor proteins. A 1% pre-culture was inoculated into fresh medium and grown until an optical density at 600 nm (OD_600_) reached 0.1 to 0.2. At this point, the expression of the prime editor was induced using an appropriate inducer, depending on the expression system employed. Following induction, the cells were electroporated with 100 ng of the epegRNA expression plasmid using 0.1-cm cuvettes at 1.8 kV, 25 µF, and 200 Ω, and were then plated onto agar plates containing the corresponding antibiotic for selection. Editing efficiency was assessed using two methods: in blue-white screening, the ratio of white to blue colonies was calculated; in the kanamycin-based positive selection, the number of colonies on kanamycin-containing plates was divided by the number of colonies on non-kanamycin plates to determine editing efficiency.

For *S. aureus*, both the prime editor and epegRNA expression plasmids were introduced via electroporation (1 µg of each plasmid at 21 kV/cm, 100 Ω, and 25 µF using a 0.1-cm cuvette; Bio-Rad). The transformed cells were cultured overnight and then plated onto agar plates containing ampicillin for selection. Individual colonies were subjected to colony PCR, and the outcomes of prime editing were confirmed by Sanger sequencing (Supplementary data_Sanger seq).

### Deep amplicon sequencing and data analysis

Genomic DNA was extracted from edited bacterial colonies collected by scraping from agar plates using the G-spin™ Genomic DNA Extraction Kit (Intron Biotechnology #17121), following the manufacturer’s protocol. The target genomic region containing the editing site was amplified in a two-step PCR process. The first PCR was performed using 100 ng of genomic DNA in a 25 µL reaction volume using Q5 High-Fidelity DNA Polymerase (New England Biolabs #M0491S). In the second PCR, indexing primers were used to add unique i7 and i5 barcodes. This reaction was performed in a 40 µL volume using 2 × Taq Smart Mix (Solgent #STD01-M50h), under the following cycling conditions: 60 °C annealing temperature, 1 min extension, and 20 cycles. PCR products were purified using the MEGAquick-spin™ Plus Total Fragment DNA Purification Kit (Intron Biotechnology #17290), and sequenced using MiSeq (Illumina). Sequencing data were analyzed using CRISPResso2 in batch mode (CRISPRessoBatch), with quantification of intended editing frequencies conducted within a ± 10 bp window centered on the predicted edit site.

### Statistical analysis

All data are expressed as the mean ± standard deviation (SD). Statistical analyses were conducted using GraphPad Prism software (version 9.4.1; GraphPad Software Inc., La Jolla, CA, USA). Group comparisons were performed using either one-way analysis of variance (ANOVA) or Student’s *t*-test, as appropriate. Statistical significance was defined as *p* < 0.05 (*p < 0.05; **p < 0.01; ***p < 0.001; ****p < 0.0001).

## Supplementary Information

Below is the link to the electronic supplementary material.

ESM 1 Supplementary data_uncropped gel for Fig_1(B)1 (PNG 74.6 KB)

High Resolution Image (TIF 2.50 MB)

ESM 2 Supplementary data_uncropped gel for Fig_1(B)2 (PNG 184 KB)

High Resolution Image (TIF 2.50 MB)

ESM3 Supplementary data_uncropped gel for Fig_S1(A) (JPG 538 KB) 

ESM 4 Supplementary data_uncropped gel for Fig_S1(B) (PNG 68.2 KB)

High Resolution Image (2.50 MB)

ESM 5 Supplementary data_uncropped gel for Fig_S6(A)1 (PNG 175 KB)

High Resolution Image (TIF 2.50 MB)

ESM 6 Supplementary data_uncropped gel for Fig_S6(A)2 (PNG 180KB)

High Resolution Image (TIF 2.50 MB)

ESM7 Supplementary data_uncropped gel for Fig_S6(B) (JPG 743 KB) 

ESM8 Supplementary data_Sanger seq (ZIP 3.38 MB) 

ESM9 Supplementary materials_5_11_2026 (PDF 2.51 MB) 

## Data Availability

All data generated during this study are available within this primary manuscript and supplementary materials.
