# Supplementary figures and images for: Adapting prime editing with split prime editors in Escherichia coli and its application to Staphylococcus aureus genome editing
Source: Appl Microbiol Biotechnol. 2026 Jun 4;110(1):212. doi: 10.1007/s00253-026-13897-9 (PMC13369037; doi:10.1007/s00253-026-13897-9)

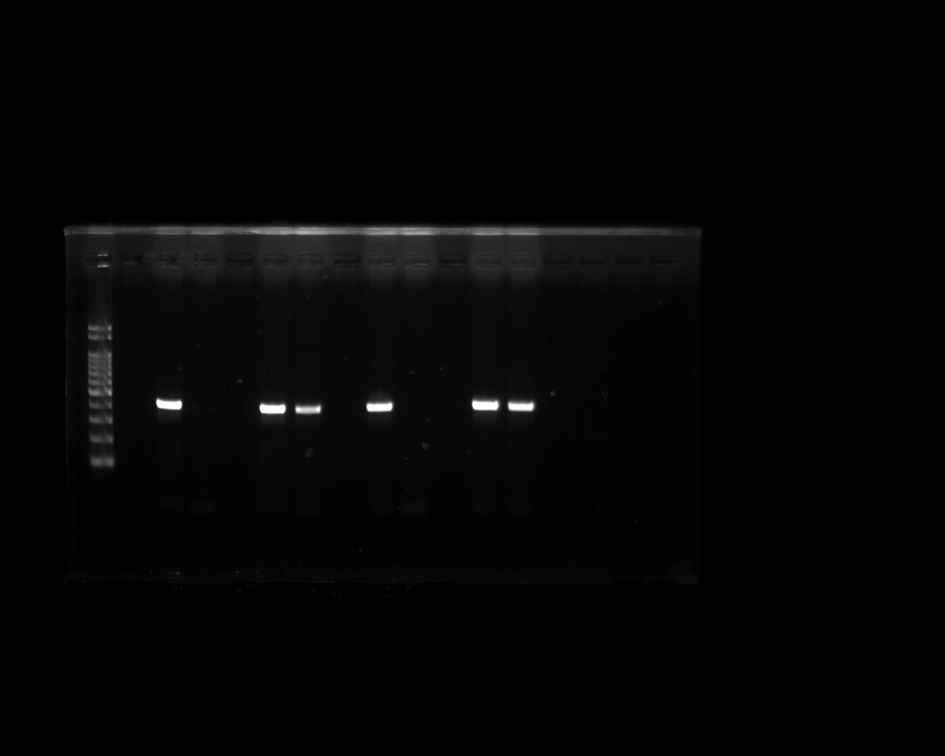

Supplement: Supplementary file 1 — Supplementary data_uncropped gel for Fig_1(B)1 (PNG 74.6 KB) [file 253_2026_13897_Fig5_ESM.png]

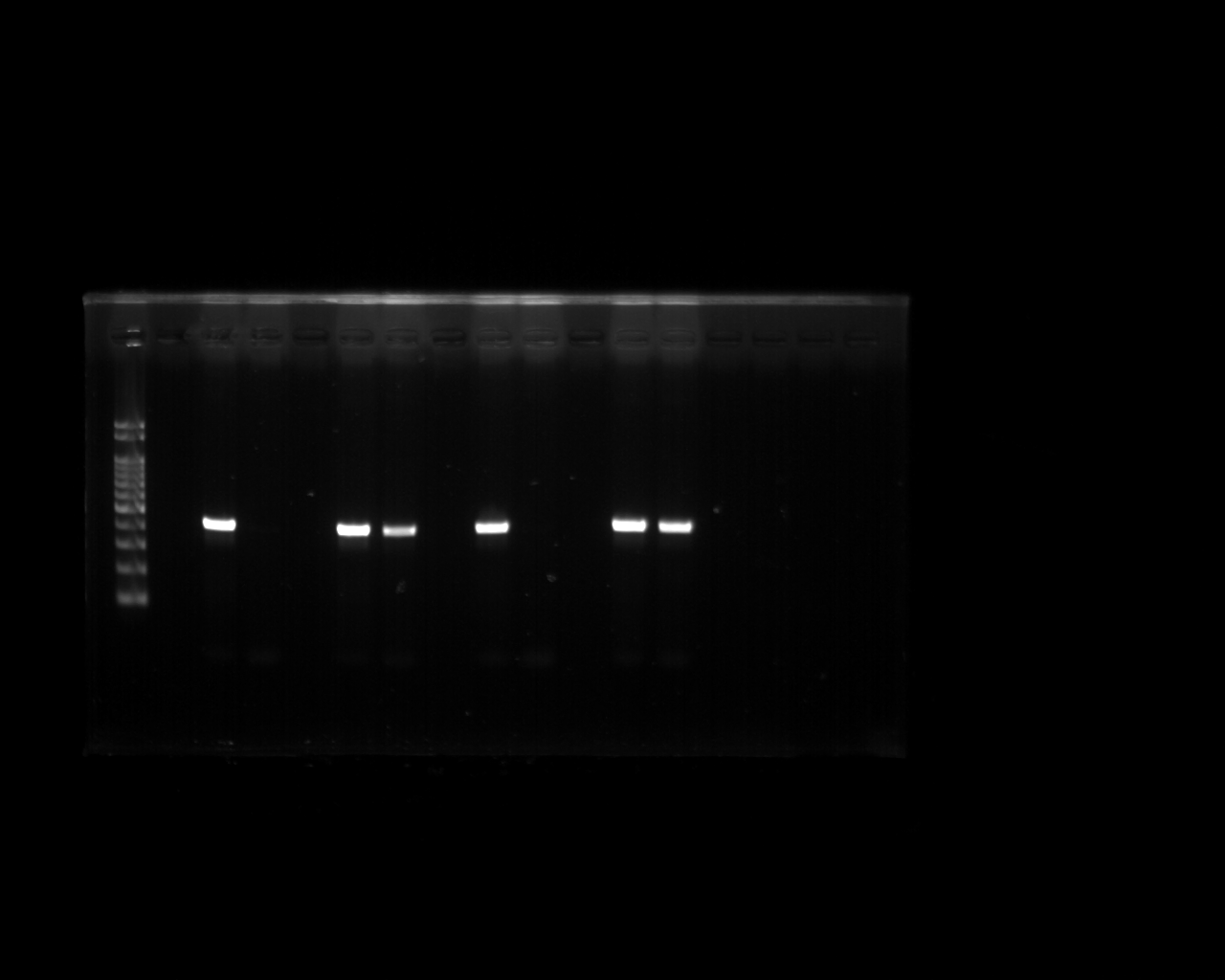

Supplement: Supplementary file 2 — High Resolution Image (TIF 2.50 MB) [file 253_2026_13897_MOESM1_ESM.tif]

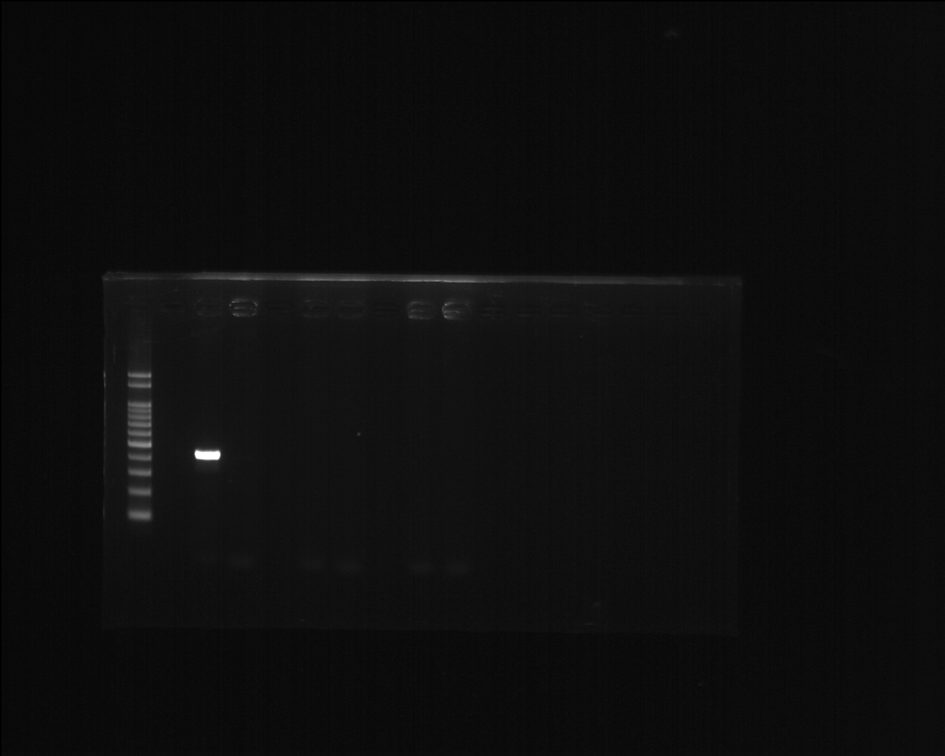

Supplement: Supplementary file 3 — Supplementary data_uncropped gel for Fig_1(B)2 (PNG 184 KB) [file 253_2026_13897_Fig6_ESM.png]

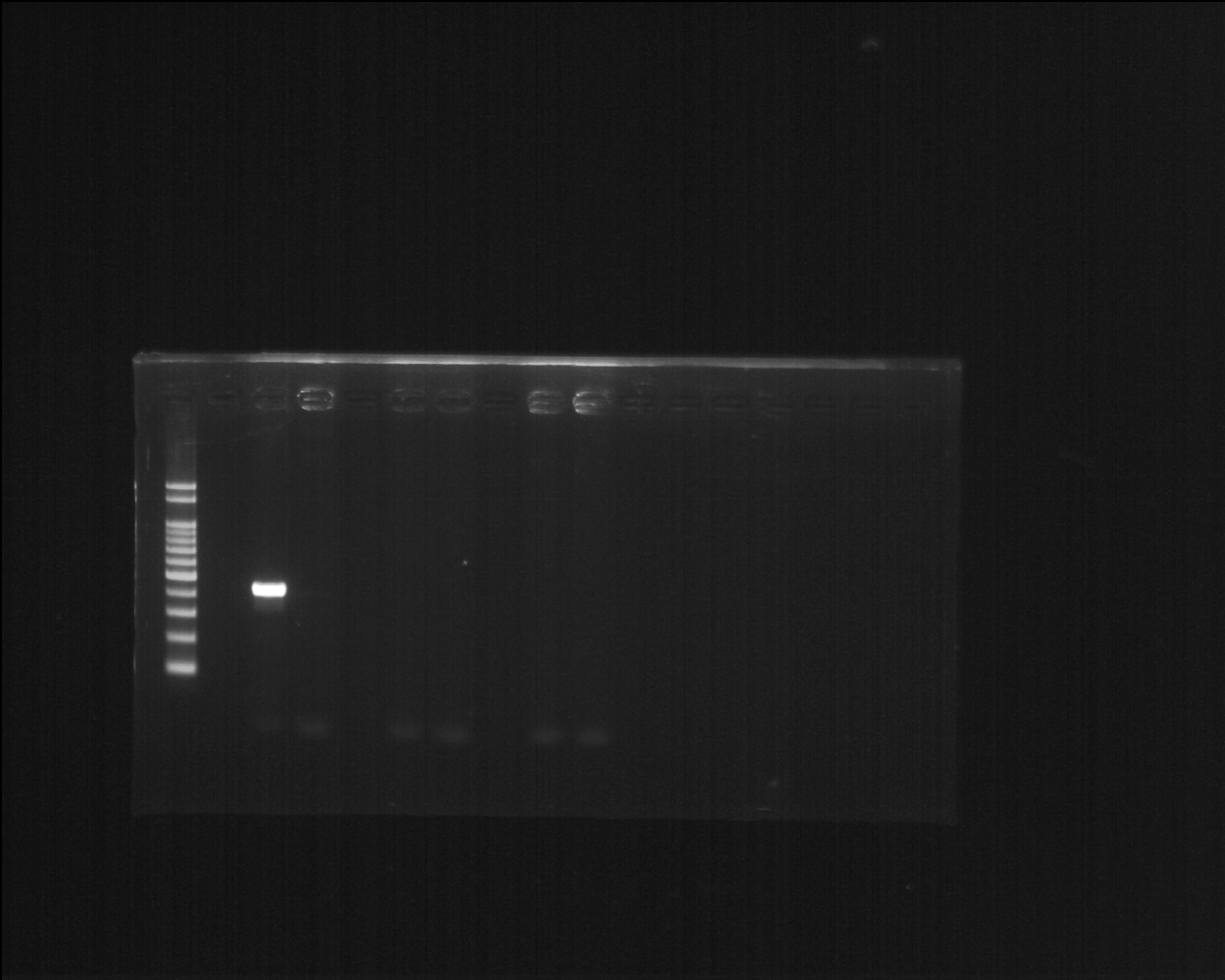

Supplement: Supplementary file 4 — High Resolution Image (TIF 2.50 MB) [file 253_2026_13897_MOESM2_ESM.tif]

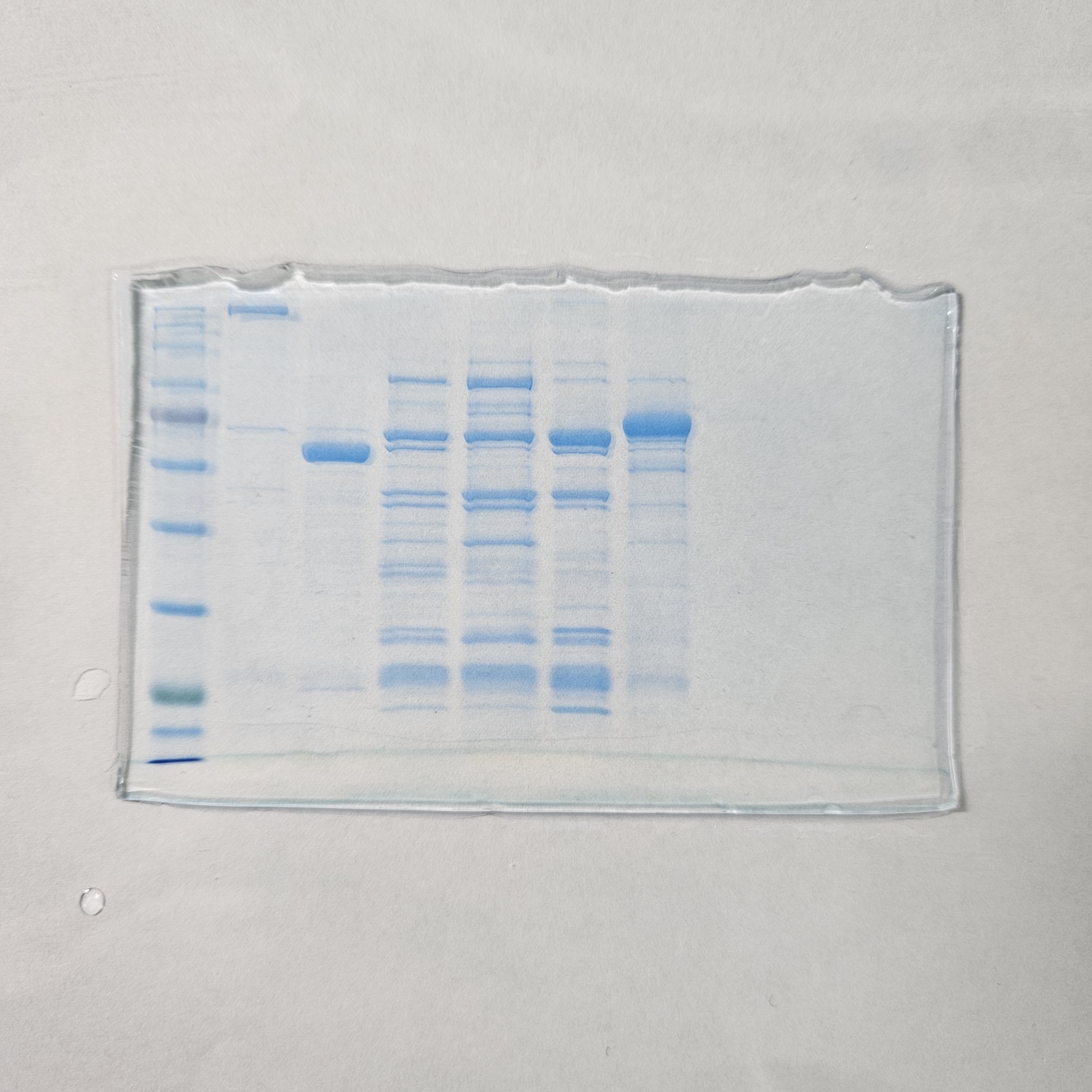

Supplement: Supplementary file 5 — Supplementary data_uncropped gel for Fig_S1(A) (JPG 538 KB) [file 253_2026_13897_MOESM3_ESM.jpg]

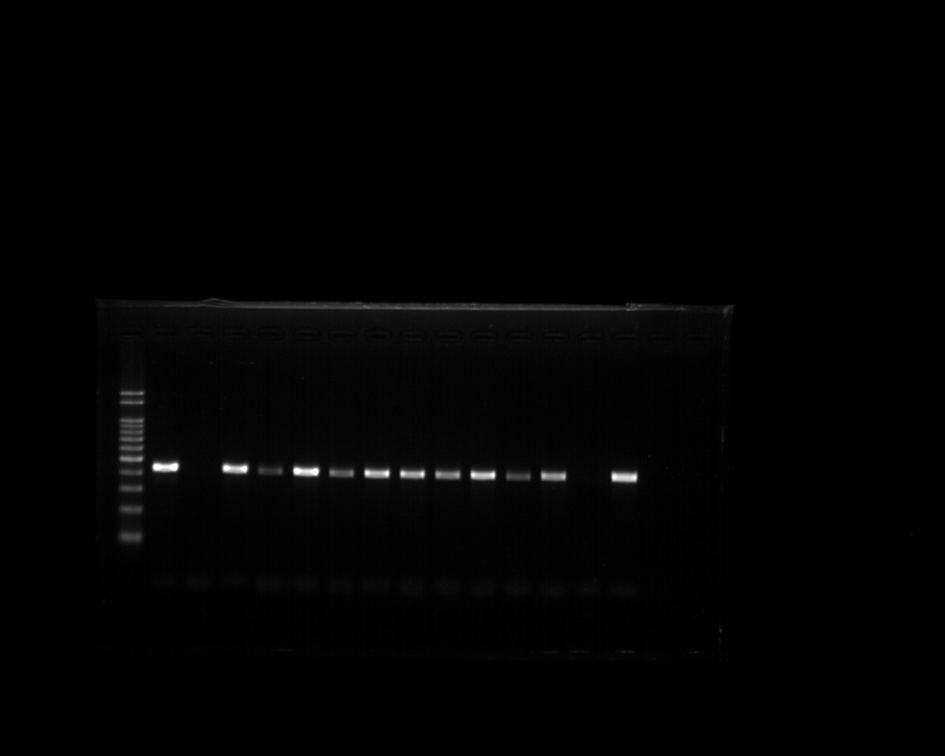

Supplement: Supplementary file 6 — Supplementary data_uncropped gel for Fig_S1(B) (PNG 68.2 KB) [file 253_2026_13897_Fig7_ESM.png]

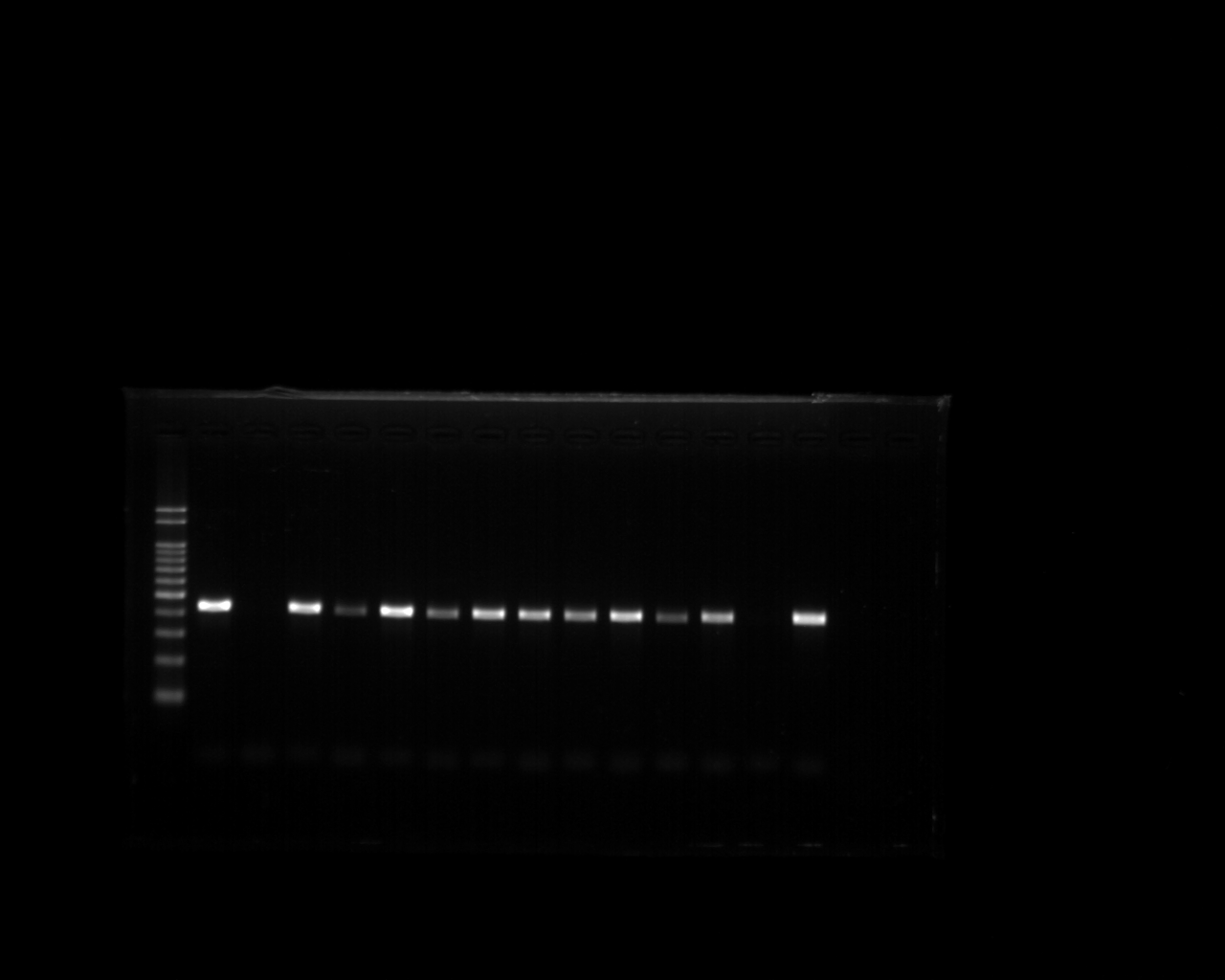

Supplement: Supplementary file 7 — High Resolution Image (2.50 MB) [file 253_2026_13897_MOESM4_ESM.tif]

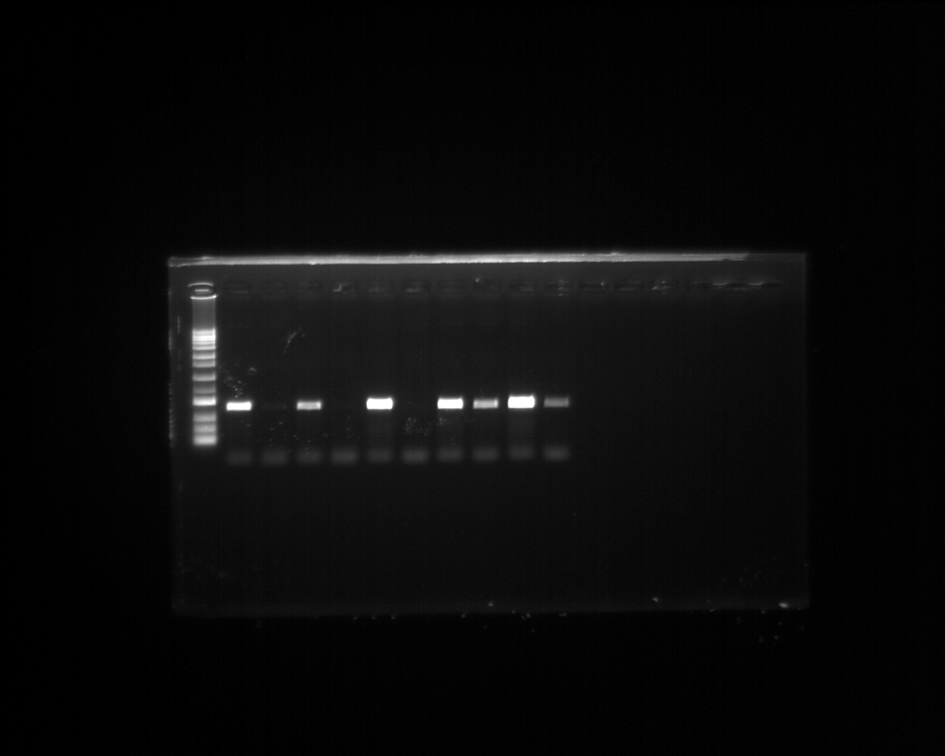

Supplement: Supplementary file 8 — Supplementary data_uncropped gel for Fig_S6(A)1 (PNG 175 KB) [file 253_2026_13897_Fig8_ESM.png]

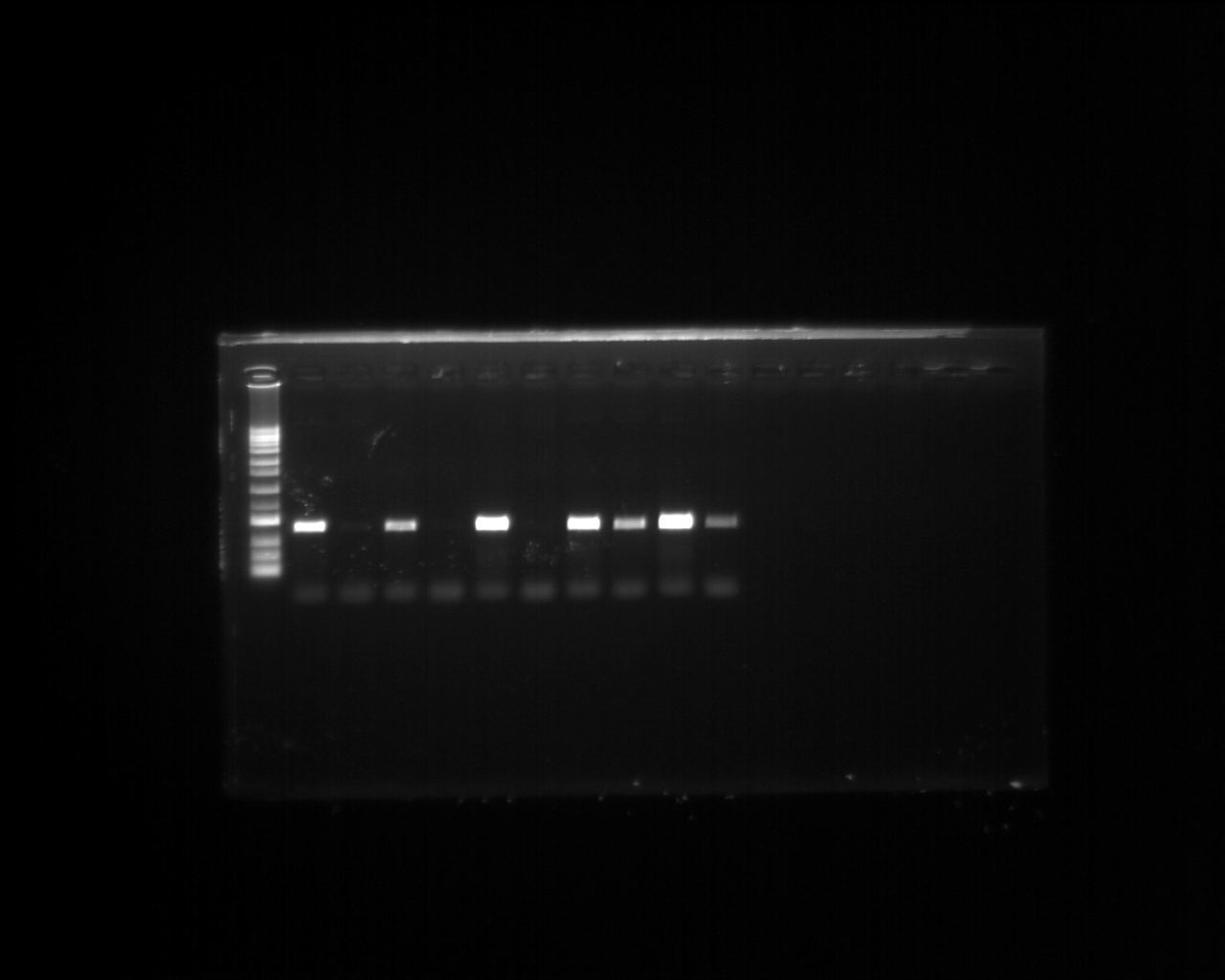

Supplement: Supplementary file 9 — High Resolution Image (TIF 2.50 MB) [file 253_2026_13897_MOESM5_ESM.tif]

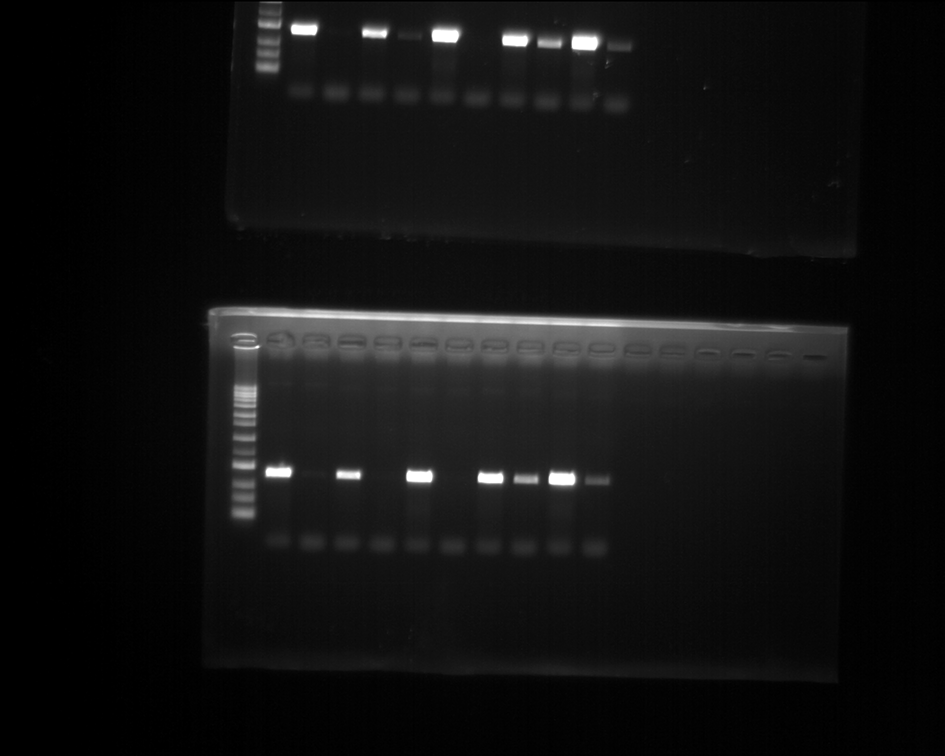

Supplement: Supplementary file 10 — Supplementary data_uncropped gel for Fig_S6(A)2 (PNG 180KB) [file 253_2026_13897_Fig9_ESM.png]

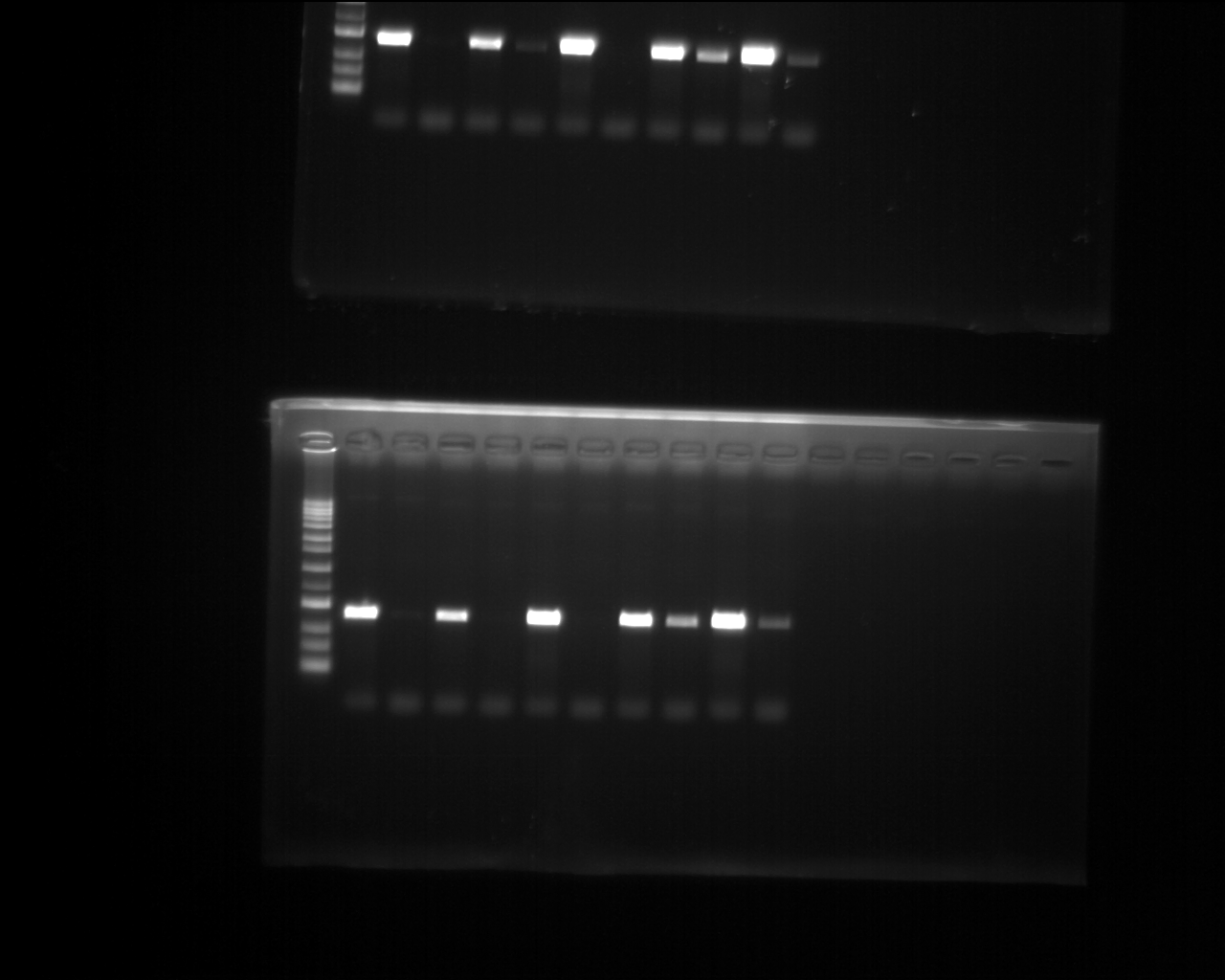

Supplement: Supplementary file 11 — High Resolution Image (TIF 2.50 MB) [file 253_2026_13897_MOESM6_ESM.tif]

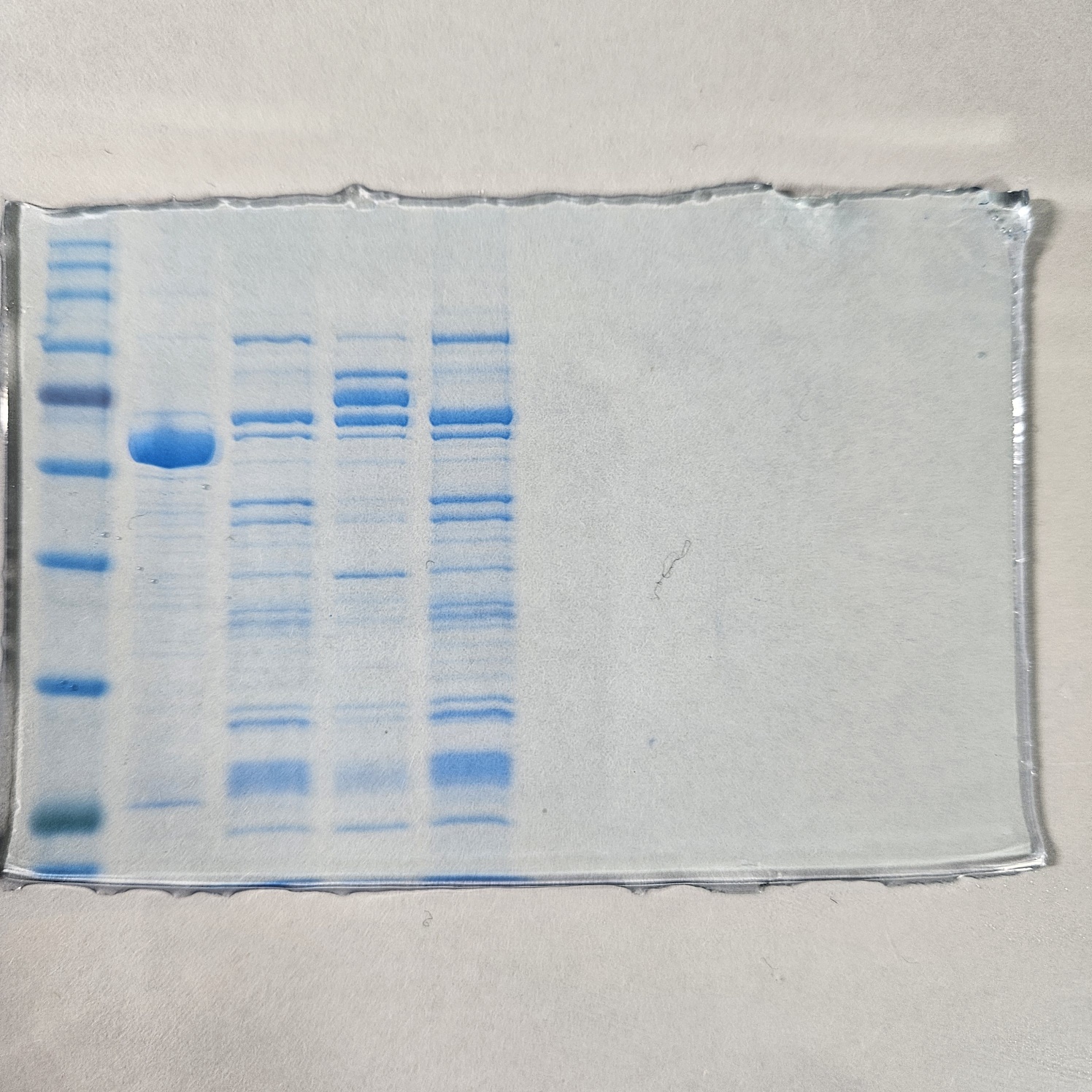

Supplement: Supplementary file 12 — Supplementary data_uncropped gel for Fig_S6(B) (JPG 743 KB) [file 253_2026_13897_MOESM7_ESM.jpg]
