# Supplementary material for: Adapting prime editing with split prime editors in Escherichia coli and its application to Staphylococcus aureus genome editing
Source: Appl Microbiol Biotechnol. 2026 Jun 4;110(1):212. doi: 10.1007/s00253-026-13897-9 (PMC13369037; doi:10.1007/s00253-026-13897-9)
Supplement: Supplementary file 14 — Supplementary materials_5_11_2026 (PDF 2.51 MB) [file 253_2026_13897_MOESM9_ESM.pdf]

`

Supplementary Materials for

Adapting Prime Editing with Split Prime Editors in *Escherichia coli* and its Application to *Staphylococcus aureus* Genome Editing

Seong Hyeok Ma[1,3,4], Goosang Yu[2,3], Suyeon Park[1,4], Hyuna Sung[1,4], Uk Jin Jeong[1,3,4], Hyongbum Henry Kim[2], Junho Cho[1,3,4*], and Sang Sun Yoon[1,3,4,5*]

This PDF file includes:

Figure S1 to S7

Tables S1 to S3

Supplementary Figure and Table Legends

Fig. S1. PE-9His purification and in vitro prime editing assay (A) SDS-PAGE analysis of purified PE2-9His. Proteins were purified using a His-tag purification method and loaded at a concentration of 2 µg. (B) PCR results based on varying concentrations of WT and pre-mutated templates (from left, negative control, to right, positive control).

Fig. S2. **Design of the epegRNA and phenotypic validation of prime editing.** (A) Illustration of the epegRNA design. The left panel shows a schematic of the epegRNA labeled with the PBS, RTT, and spacer. The right panel describes the targeted locus with the corresponding components of the spacer, RTT, PBS, and linker. (B) Representative images of white and blue colonies. The left panel shows representative blue and white colonies on an agar plate, and the right panel shows the streaks of the confirmed white and blue colonies for further verification.

Fig. S3. Phenotypic validation of prime editing in MRSA via antibiotic selection. Both plates contain ampicillin to select for *mecA*-reverted clones following a 1-ml overnight enrichment. The left panel shows the negative control lacking the epegRNA, where no colonies were observed after plating 100 µl of a 10-fold diluted sample. The right panel demonstrates the robust growth of successfully edited colonies in the presence of the epegRNA, even after plating 100 µl of a 1,000-fold diluted sample.

Fig. S4. Prime editing with SaPE2 and SaPE2ΔRH in *E. coli MG1655* Δ*sbcB*, Δ*exoX*, Δ*xseA*. (A) illustration of SpPE2 and SaPE2. (B) Results of prime editing targeting *lacZ* and Kan^RΔ66-67 with SaPE2 and SaPE2ΔRH. Data represent mean ± s.d. of n = 3 independent replicates.

Fig. S5. Optimization of prime editing in in *E. coli* MG1655*.* (A) Comparison of Efficiency according to knock out of key exonucleases known to inhibit prime editing through previous studies. Data represent mean ± s.d. of n = 3 independent replicates. (B) Schematic of Kanamycin selection. (C) optimization of inducers concentration for untethered PE system. Data represent mean ± s.d. of n = 3 independent replicates.

`

Fig. S6. Prime editing with purified SpPE2 and pegRNAs. (A) Result of prime editing with purified SpPE2. Purified PE2-9His were transformed to *E. coli* MG1655 harboring pePEgRNA(opti)_KanR_+66-67_TG ins. The experimental conditions varied depending on the amount of purified PE2-9His, the presence or absence of sucrose, and the electroporation voltage. (B) Result of prime editing with purified pegRNAs. Purified epegRNA_KanR_+66-67_TG ins were transformed to *E. coli* MG1655 harboring pCRISPR_PE. The experimental conditions varied depending on the amount of purified PE2-9His, the presence or absence of sucrose and salt. Additional lower-molecular-weight bands in C-Cas9 and RT lanes are likely due to partial proteolytic cleavage and the limitation of single-step Ni-NTA purification.

Fig. S7. In vitro prime editing assay with purified split PE. (A) PCR results with split PE composed of purified N-Cas9(535N-GCN4), C-Cas9 (536C_GCN4), RT using His-tag purification. PE2-9His was used instead of split PE, as a positive control of in vitro prime editing g assay.

(B) SDS-PAGE analysis of purified split PE. Proteins were purified using a His-tag purification method and loaded at a concentration of 3 µg. The red arrows indicate each of the purified proteins.

Table. S1. pegRNAs used in this study.

Table. S2. Plasmids used in this study.

Table. S3. Primers used in this study.

Supplementary Figure. 1

(A)

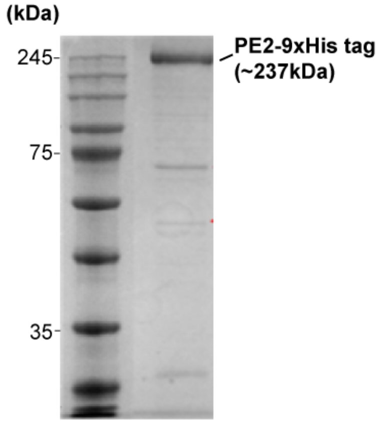

(B)

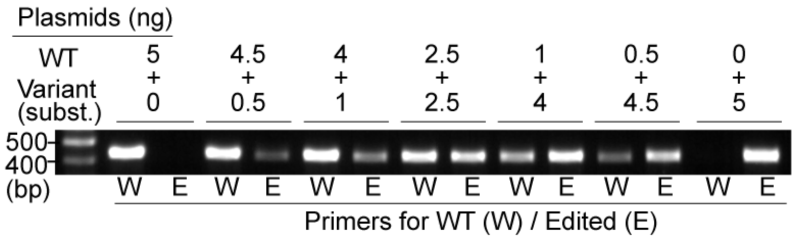

Supplementary Figure. 2

(A)

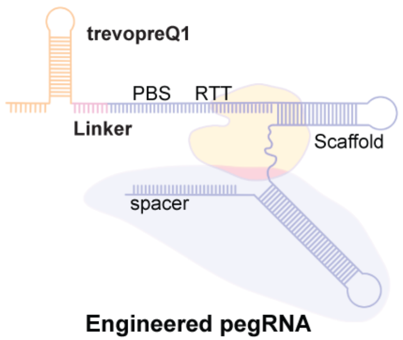

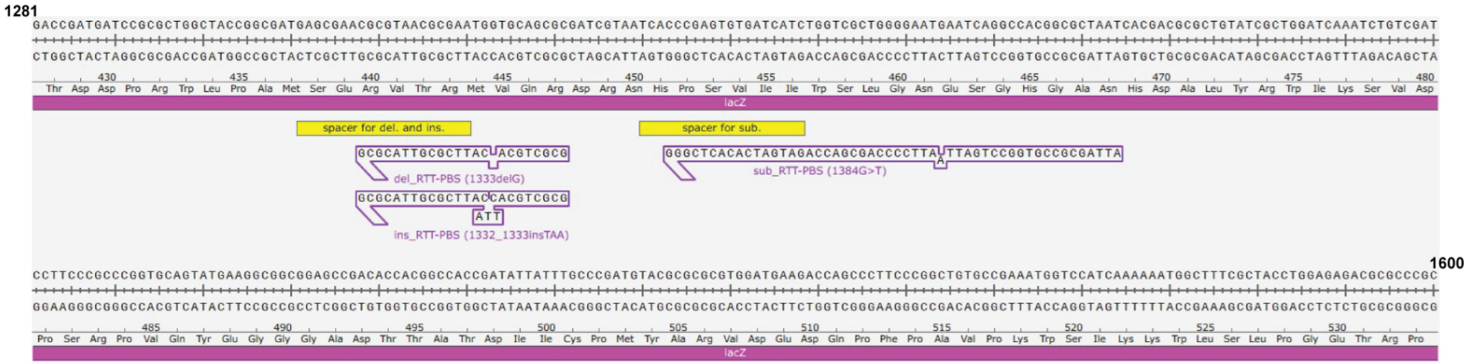

pegRNA	Spacer	RTT	PBS	Linker
1384G>A	ATCACCCGAGTGTGATCATC	ATTAGCGCCGTGGCCTGATTAATTCCCCAGCGACCAGAT (39bp)	GATCACACTCGGG (13bp)	ACAATAAG
1333delG	GAGCGAACGCGTAACGCGAA	GCGCTGCACATTC (13bp)	GCGTTACGCG (10bp)	ACAATAAG
1332_1333insTAA	GAGCGAACGCGTAACGCGAA	GCGCTGCACTTACATT(16bp)	CGCGTTACGCG (11bp)	ACAATAAG

(B)

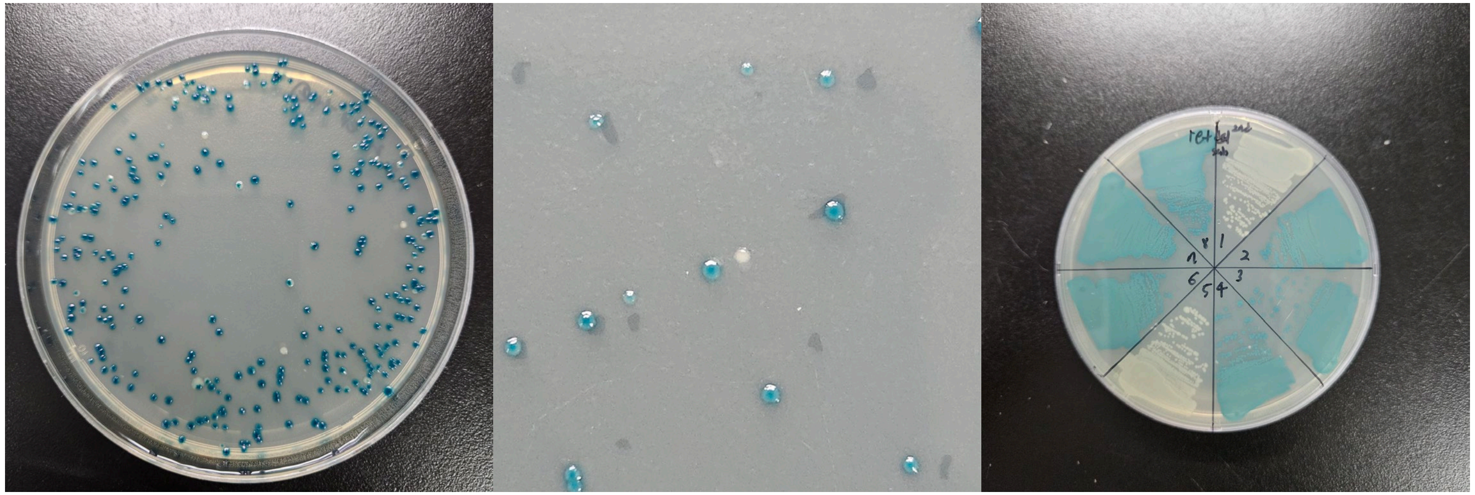

Supplementary Figure. 3

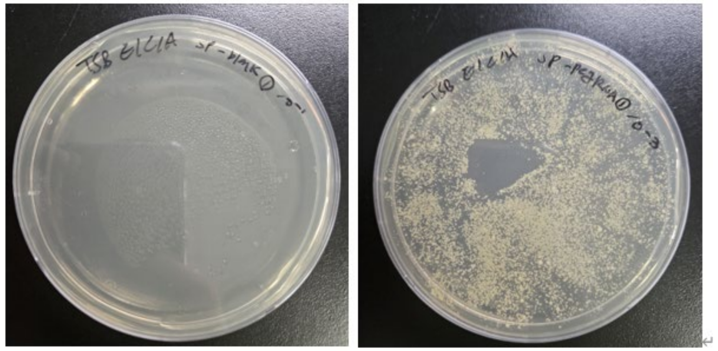

Without pegRNA With pegRNA

Supplementary Figure. 4

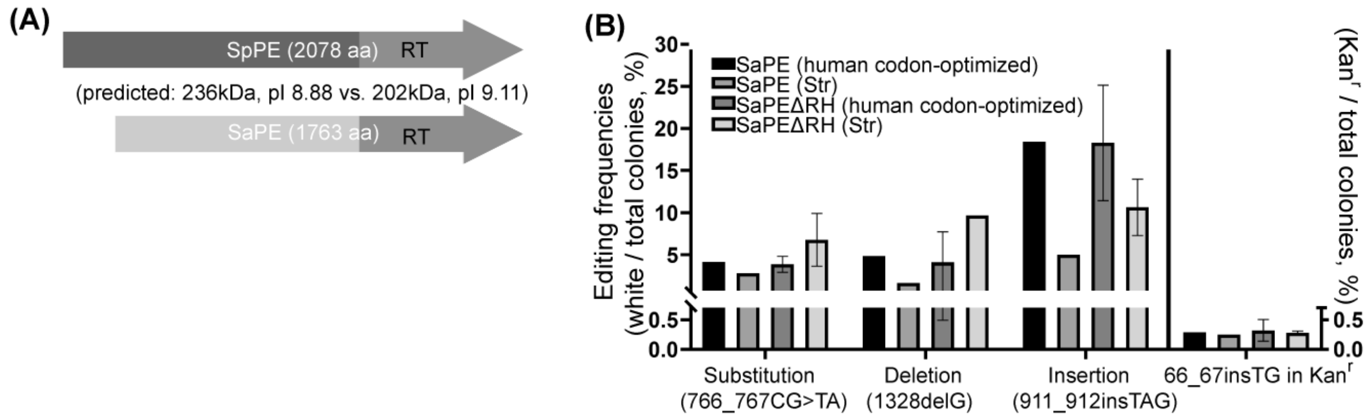

Supplementary Figure. 5

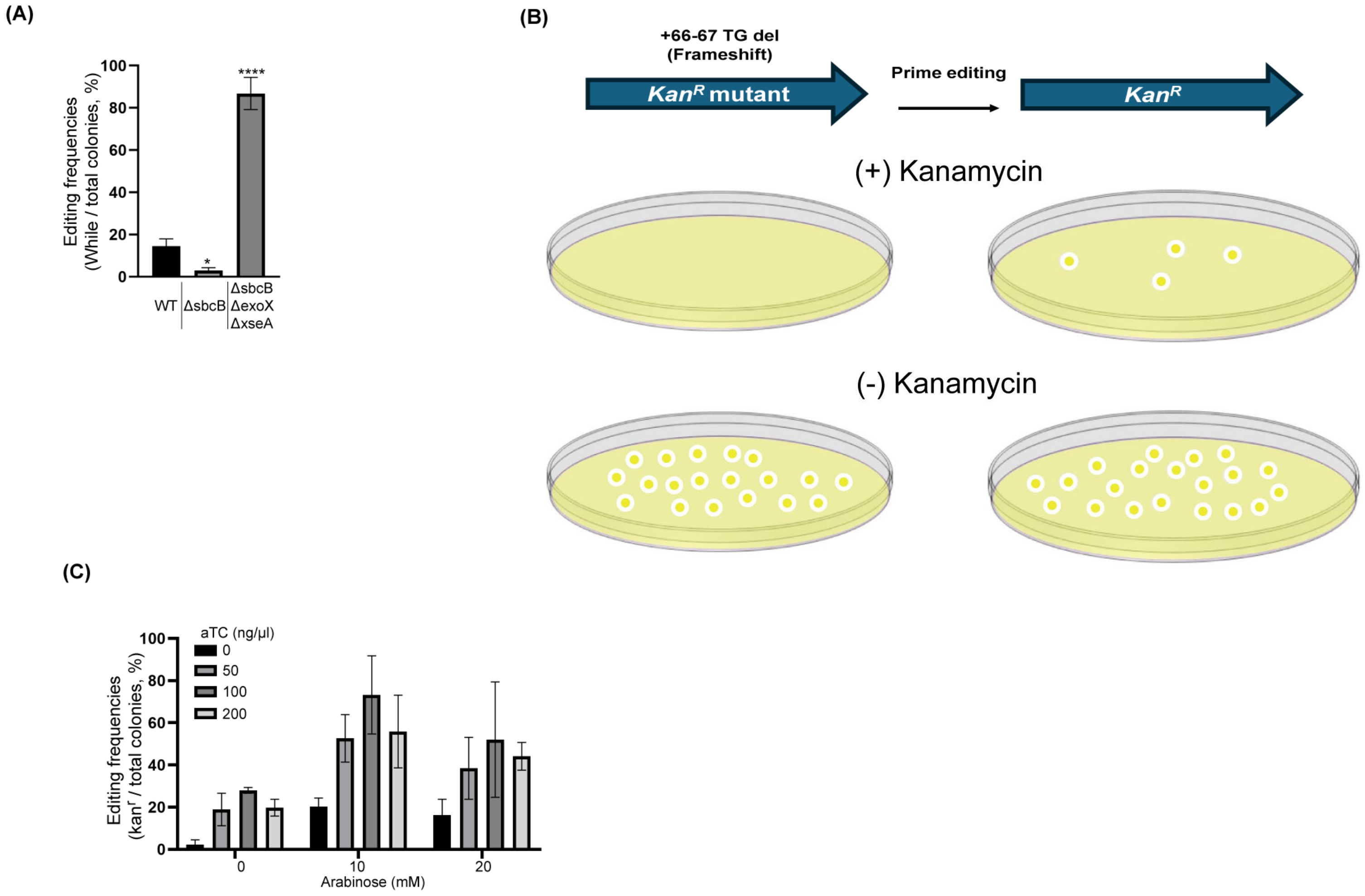

Supplementary Figure. 6

(A)

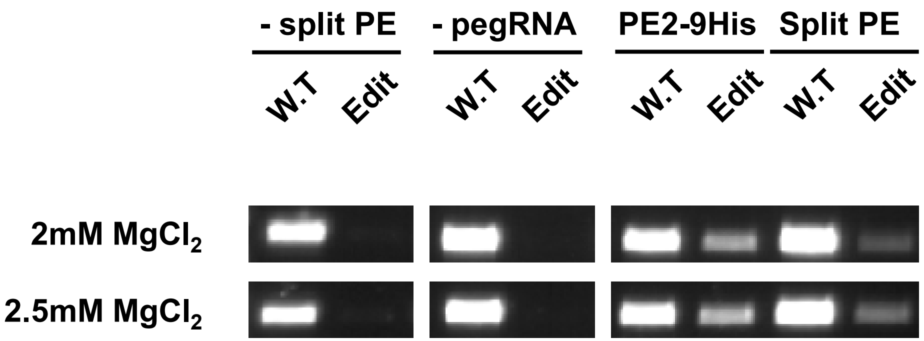

(B)

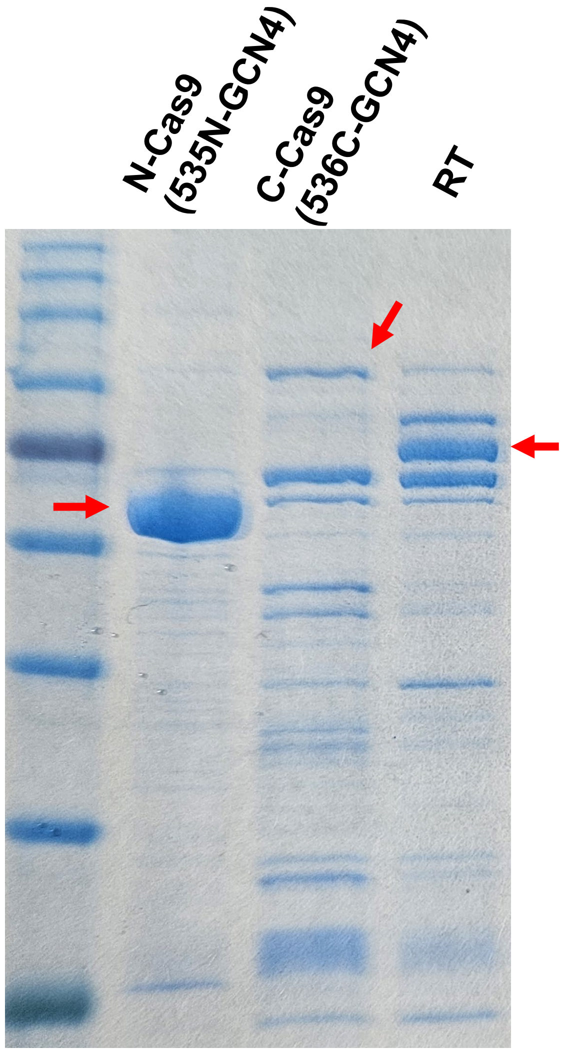

Supplementary Figure. 7

(A)

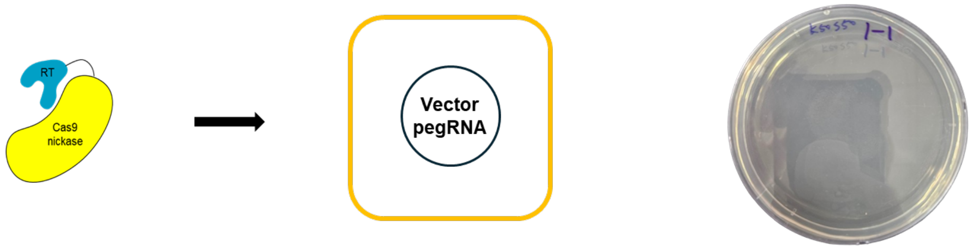

	Sucrose	condition	Time constant
PE-1ul	X	Ec2(2.5kV)	5.4
PE-1ul	O	Ec2	5.3
PE-3ul	O	Ec2	4.7
PE-5ul	O	Ec2	3.8
PE-1ul	O	Ec1(1.8kV)	4.5

(B)

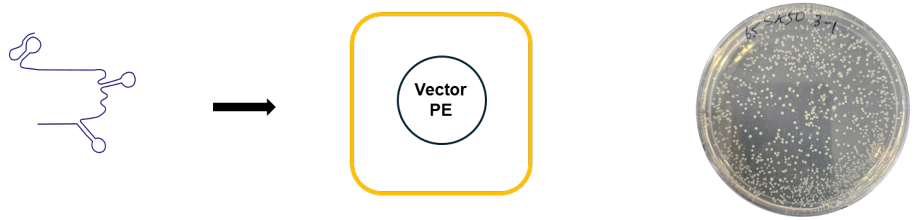

	PE	Sucrose	Salt	Time constant after electroporation	CFU on Kan plate	Total CFU
+66-67<br>+281-282	X	X	X	6<br>6	561<br>219	4.24 x 10^6<br>5.92 x 10^6
+66-67<br>+281-282	X	O	X	6<br>6	360<br>118	3.84 x 10^6<br>8.64 x 10^6
+66-67<br>+281-282	O	O	X	5.2<br>5.2	93<br>31	2.32 x 10^6<br>4.64 x 10^6
+66-67<br>+281-282	O	O	O	3.4<br>3.2	8<br>0	2.24 x 10^6<br>2.48 x 10^6

Table. S1. pegRNAs used in this study.

pegRNA	Spacer	Scaffold	RTT	PBS	Linker	Deep Prime score
lacZ_+763-764_CTtoTAsub	GCGAGTTGCGTGACTACCTA	Conventional	ACTGTTACCCGTTA (14bp)	GTAGTCACGCAACT (14bp)	TATCATCC	24.13
lacZ_+631-632_CGdel	TATGCAGCAACGAGACGTCA	Conventional	ATGAGCGGCATTTTCTGA (18bp)	CGTCTCGTTGCTGC (14bp)	CCTACACC	8.76
lacZ_+936-937_TAGins	AATCCCGAATCTCTATCGTG	Conventional	TCAACCACCTACGCAC (16bp)	GATAGAGATTCGG (13bp)	ACAACATA	24.27
lacZ_+1384_GtoTsub	ATCACCCGAGTGTGATCATC	Optimized	ATTAGCGCCGTGGCCTGATTAATTCCCCAGCGACCAGAT (39bp)	GATCACACTCGGG (13bp)	ACAATAAG	39.51
lacZ_+1333_Gdel	GAGCGAACGCGTAACGCGAA	Optimized	GCGCTGCACATTC (13bp)	GCGTTACGCG (10bp)	ACAATAAG	41.93
lacZ_+1332-1333_TAGins	GAGCGAACGCGTAACGCGAA	Optimized	GCGCTGCACTTACATT (16bp)	CGCGTTACGCG (11bp)	ACAATAAG	42.39
KanR_+66-67_TGins	GGAGAGGCTATTCGGCTAAC	Optimized	TGTGCCCAGTCAT (13bp)	AGCCGAATAGCC (12bp)	AGAAATAG	44.28
KanR_+281-282_AGdel	AAGGGACTGGCTGCTATAGT	Optimized	GGCACTTCGCCCA (13bp)	ATAGCAGCCAGTC (13bp)	GAATGATC	42.53
mecA_+56-57_AAtoTTsub	ATAGTTGTAGTTGTCGGGTA	Optimized	AAATATATACCAAAC (15bp)	CCGACAACTAC (11bp)	TCTCAACG	24.71
pegRNA	Spacer	Scaffold	RTT	PBS	Linker	-
(Sa)_lacZ_+766-767_CGtoTAsub	CGGCGAGTTGCGTGACTACCT	SaCas9 scaffold	ACTGTTACCTATAG (14bp)	GTAGTCACGCAAC (13bp)	ATATTATC	-
(Sa)_lacZ_+1328 Gdel	GGCGATGAGCGAACGCGTAAC	SaCas9 scaffold	GCACCATTGCGTT (13bp)	ACGCGTTCGCT (11bp)	AAATAATA	-
(Sa)_lacZ_+921-922_TAGins	AAACTGTGGAGCGCCGAAATC	SaCas9 scaffold	GATAGAGATTCTACGGGAT (19bp)	TTCGGCGCTCC (11bp)	AAATTAAG	-
(Sa)_KanR_+66-67_TGins	TTGTCTGTTGTGCCCAGTTAG	SaCas9 scaffold	GGCTATTCGGCTATG (15bp)	ACTGGGCACAACA (13bp)	TAATCTCC	-
(Sa) mecA_+56-57_AAtoTTsub	ttattttaatagttgtagttg	SaCas9 scaffold	TATATACCAAAC (12bp)	CCGACAACTAC (11bp)	ATATTATC	-

`

Table. S2. Strains and Plasmids used in this study.

Strains

Name	Discription	source
DH5α	for Plasmid construction	This study
ArcticExpress RIL (DE3)	*E. coli* B F^{-} *ompT hsdS*($r_B{}^{-}$ $m_B{}^{-}$) *dcm*$^{+}$ Tet^{r} *gal* λ(DE3) *endA* Hte [*cpn10 cpn60 Gent*^{r}] [*argU ileY leuW Str*^{r}]	Agilent Technologies
MG1655	K12 F- lambda- ilvG- rfb-50 Rh-1	lab stock
MG1655 ΔsbcB	Knock out of *sbcB* for increasing prime editing efficiency	This study
MG1655 ΔsbcB ΔxseA ΔexoX	Knock out of *sbcB, xseA, exoX* for increasing prime editing efficiency	This study
MG1655 ΔybcC::kan[TG66-67del]	Replacement of *ybcC* to *Kan*^{R} containing +66-67 TG deletion	This study
JE2	*Staphylococcus aureus* USA300 LAC JE2	lab stock
JE2 mecA[TTtoAA56-57Sub]	JE2 ccontaining +56-57 TT to AA mutation in *mecA*	This study

Plasmids

Name	Discription	source
pCRISPR_PE bacteria	Harboring SpPE2	Addgene #132730
pPEgRNA	Backbone of pegRNA	Addgene #172716
pCDF-GF plus	Backbone of RT variants	Addgene ##172718
Topo_Opti scaffold	Harboring opti-scaffold	
pLenti-BSD	Harboring SaPE2(Human)	
pCRISPomyces-SaCas9	Harboring SaPE2(STR)	Addgene #129553
pET21b	Backbone of	lab stock
pBAD24	Backbone of nSpCas9	lab stock
pVIK112	Template of in vitro prime editing assay	lab stock
pCas	For consturction of E. coli MG1655 mutants	Addgene #62225
pTargetF	For consturction of E. coli MG1655 mutants	Addgene #62226
pMAD	For consturction of MRSA JE2 mutant	https://pubmed.ncbi.nlm.nih.gov/15528558/, lab stock
pEPSA5	For consturction of MRSA JE2 mutant	https://onlinelibrary.wiley.com/doi/full/10.1046/j.1365-2958.2002.02832.x?sid=nlm%3Apubmed, lab stock

`

pSSY01	Cloning of tevopreQ1 into pPEgRNA	In this study
pSSY02	Cloning of opti-scaffold into pSSY01	In this study
pSSY03	Replacement of J23119 promoter to T7 promoter in pSSY01 for in vitro transcription	In this study
pSSY04	Cloning of lacZ_+1384_GtoTsub pegRNA into pSSY03	In this study
pSSY05	substitution of G to T in the lacZ gene within pVIK112	In this study
pSSY06	Cloning of SpPE2-9His gene into pET21b	In this study
pSSY07	Cloning of lacZ_+763-764_CTtoTAsub into pPEgRNA	In this study
pSSY08	Cloning of lacZ_+631-632_CGdel into pPEgRNA	In this study
pSSY09	Cloning of lacZ_+936-937_TAGins into pPEgRNA	In this study
pSSY10	Cloning of lacZ_+763-764_CTtoTAsub into pSSY01	In this study
pSSY11	Cloning of lacZ_+631-632_CGdel into pSSY01	In this study
pSSY12	Cloning of lacZ_+936-937_TAGins into pSSY01	In this study
pSSY13	Cloning of lacZ_+1384_GtoTsub into pSSY02	In this study
pSSY14	Cloning of lacZ_+1333_Gdel into pSSY02	In this study
pSSY15	Cloning of lacZ_+1332-1333_TAGins into pSSY02	In this study
pSSY16	Cloning of KanR_+66-67_TGins into pSSY02	In this study
pSSY17	Cloning of KanR_+281-282_AGdel into pSSY02	In this study
pSSY18	deletion of RnaseH domain in pCRISPR-PE bacteria	In this study
pSSY19	deletion of N-terminal part in pSSY18	In this study
pSSY20	Cloning of nSpCas9 into pBAD24 and replacement of Amp^R to Cm^R	In this study
pSSY21	Replacement of J23109 promoter to tetracycline inducible promoter with tetR in pCDF-GFlus	In this study
pSSY22	Cloning of MMV-RT into pSSY21	In this study
pSSY23	Cloning of MMV-RTΔRH into pSSY21	In this study
pSSY24	Cloning of MMV-RT mini into pSSY21	In this study
pSSY25	Deletion of part of nSpCas9 for N-nSpCas9(573N)	In this study
pSSY26	Deletion of part of nSpCas9 for N-nSpCas9(674N)	In this study
pSSY27	Deletion of part of nSpCas9 for N-nSpCas9(713N)	In this study

`

pSSY28	Deletion of part of nSpCas9 for C-nSpCas9(574C)	In this study
pSSY29	Deletion of part of nSpCas9 for C-nSpCas9(675C)	In this study
pSSY30	Deletion of part of nSpCas9 for C-nSpCas9(714C)	In this study
pSSY31	Combination of pSSY25 and pSSY28	In this study
pSSY32	Combination of pSSY26 and pSSY29	In this study
pSSY33	Combination of pSSY27 and pSSY30	In this study
pSSY34	Cloning of gp41-1 intein into pSSY31	In this study
pSSY35	Cloning of gp41-1 (+) intein into pSSY31	In this study
pSSY36	Cloning of NrdJ-1 intein into pSSY31	In this study
pSSY37	Cloning of NrdJ-1(+) intein into pSSY31	In this study
pSSY38	Cloning of P3-P4 heterodimer into pSSY31	In this study
pSSY39	Cloning of gp41-1 intein into pSSY32	In this study
pSSY40	Cloning of gp41-1 (+) intein into pSSY32	In this study
pSSY41	Cloning of NrdJ-1 intein into pSSY32	In this study
pSSY42	Cloning of NrdJ-1(+) intein into pSSY32	In this study
pSSY43	Cloning of P3-P4 heterodimer into pSSY32	In this study
pSSY44	Cloning of gp41-1 intein into pSSY33	In this study
pSSY45	Cloning of gp41-1 (+) intein into pSSY33	In this study
pSSY46	Cloning of NrdJ-1 intein into pSSY33	In this study
pSSY47	Cloning of NrdJ-1(+) intein into pSSY33	In this study
pSSY48	Cloning of P3-P4 heterodimer into pSSY33	In this study
pSSY49	Cloning of SaCas9 gRNA sacffold into pSSY01	In this study
pSSY50	Cloning of (Sa)_lacZ_+766-767_CG toTAsub into pSSY49	In this study
pSSY51	Cloning of (Sa)_lacZ_+1328 Gdel into pSSY49	In this study
pSSY52	Cloning of (Sa)_lacZ_+921-922_TAGins into pSSY49	In this study
pSSY53	Cloning of (Sa)_KanR_+66-67_TG ins into pSSY49	In this study
pSSY54	Replacement of part of nSpCas9 to nSaCas9 from pCIRSPomyces-SaCas9 in pCRISPR-PE bacteria	In this study
pSSY55	Replacement of part of nSpCas9 to nSaCas9 from pCIRSPomyces-SaCas9 in pSSY18	In this study

`

**pSSY56**	**Replacement of part of nSpCas9 to nSaCas9 from pLenti-BSD in pCRISPR-PE bacteria**	**In this study**
**pSSY57**	**Replacement of part of nSpCas9 to nSaCas9 from pLenti-BSD in pSSY18**	**In this study**
**pSSY58**	**Cloning of gRNA targeting *sbcB* into pTargetF**	**In this study**
**pSSY59**	**Cloning of gRNA targeting *exoX* into pTargetF**	**In this study**
**pSSY60**	**Cloning of gRNA targeting *xseA* into pTargetF**	**In this study**
**pSSY61**	**Cloning of gRNA targeting *ybcC* into pTargetF**	**In this study**
**pSSY62**	**Replacement of bgaB to SpPE2**	**In this study**
**pSSY63**	**Replacement of bgaB to SaPE2**	**In this study**
**pSSY64**	**Cloning of split nSpCas9 (573N/574C-NrdJ-1(+)) under the control of *clpB* promoter and *sarA* promoter**	**In this study**
**pSSY65**	**Cloning of *rrnB* promoter into pEPSA5**	**In this study**
**pSSY66**	**Cloning of SpPE2 cognate pegRNA targeting mecA into pSSY63**	**In this study**
**pSSY67**	**Cloning of SaPE2 cognate pegRNA targeting mecA into pSSY63**	**In this study**
**pSSY68**	**Cloning of RTΔRH into pSSY66**	**In this study**
**pSSY69**	**Cloning of mecA_+56-57 TT to AA casstte into pMAD**	**In this study**

`

Table. S3. Primers used in this study.

Name	Sequence
pSSY06	
PE_O/E_seq_F1	AAA ATC TCA TTG CTC AGC TCC
PE_O/E_seq_F2	GGA ATG CGA AAA CCA GC
PE_O/E_seq_F3	TTG AGT GAA CTT GAT AAA GCT G
PE_O/E_seq_F4	GCT CTG CCA AGC AAA TAT GTG
PE_O/E_seq_F5	TGC TGG ATC TGA AGG ACG C
PE_O/E_F1	GTG GAC AGC AAA TGG GTC GGA TGG ATA AGA AAT ACT CAA TAG G
PE_O/E_R1	AAA ATC TTT ATC TTT AAT AAT TTT TAG CAA ATC ATG GTA G
PE_O/E_F2	AAT TAT TAA AGA TAA AGA TTT TTT GGA TAA TGA AG
PE_O/E_R2	TCT CTA TGT TTG TTA TAT GCA CTA AGA ACT TTA TC
PE_O/E_F3	GCA TAT AAC AAA CAT AGA GAC AAA CCA ATA C
PE_O/E_R3	AGT GGT GGT GGT GGT GGT GCT TCG GGC TGC TAT TTT CAA
SpPE2-RT varaints	
RH_F1	TAA CTC GAG TAA GGA TCT CCA GG
mini_F1	GAT CTA AAG AGG AGA AAG GAT CTA TGG
mini_F2	TTG ATT TGA GTC AGC TAG GAG GTG AC
mini_F3	AGC GGC GGC AGC AGC GGC GGC AGC AGC GGC AGC GAA ACC CCG GGC ACC AGC GAA AGC GCG ACC CCG GAA
mini_F4	AGC AGC GGC GGC AGC AGC GGC GGT AGC AGC CTG GGT AGC ACC TGG CTG AGC G
mini_F5	GAA CGC TCT CTA CTA GAG TC
mini_R1	GTC ACC TCC TAG CTG ACT CAA ATC
mini_R2	GCT GCC GCT GCT GCC GCC GCT GCT GCC GCC GCT GTC ACC TCC TAG CTG ACT CAA ATC
mini_R3	GCT GCT ACC GCC GCT GCT GCC GCC GCT GCT TTC CGG GGT CGC GCT TTC GCT GGT GCC CGG GGT TTC
mini_R4	CTC AGC CAG GTG CTA CCC AG
untethered PEs	
Split_F1	GCG AAG ATA ATG GTG TGT GAC CCG TGC CAG GCA TCA AAT AAA ACG
Split_F2	CCG ATT GGT GGT TCG TCC AGA AGG TCT CAA CGA TAT TTC TCC TCT TTA ATC TTC ATT GAT GGA GAA ACA GTA GAG AGT TG
Split_R1	CTG GAC GAA CCA CCA ATC GGT CTG GTC TCA AGT GTA TTT CTC CTC TTT AAT CTC TAG TAA TGG AGA AAC AGT AGA GAG TTG

`

**Split_R2**	**ACG GGT CAC ACA CCA TTA TCT TCG CAA GAA ACC AAT TGT CCA TAT TGC**
**Split_Sp_F1**	**CGA AGA TAA TGG TGT GTG AC**
**Split_Sp_R1**	**AGT GTA TTT CTC CTC TTT AAT CTC**
**Split_Sp_F2**	**AGA GAT TAA AGA GGA GAA ATA CAC TAT GGA TAA GAA ATA CTC AAT AGG CTT AG**
**Split_Sp_R2**	**ACG GGT CAC ACA CCA TTA TCT TCG TTA GTC ACC TCC TAG CTG ACT CAA ATC**
**tet_RT_F1**	**ATG ACC CTG AAC ATC GAG G**
**tet_RT_F2**	**GCA ATT TAT CTC TTC AAA TGT AGC ATT AAG ACC CAC TTT CAC ATT TAA G**
**tet_RT_R1**	**TGC TAC ATT TGA AGA GAT AAA TTG C**
**tet_RT_R2**	**TCG TCC TCG ATG TTC AGG GTC ATA GAT CCT TTC TCC TCT TTA GAT C**
**tet_RTΔRH_F1**	**TAA TAA CTC GAG TAA GGA TCT CCA GG**
**tet_mini_F1**	**TAA TAA CTC GAG TAA GGA TCT CC**
**tet_mini_R1**	**CAT AGA TCC TTT CTC CTC TTT AG**
**tet_mini_F2**	**CTG GGT AGC ACC TGG CTG AGC G**
**tet_mini_R2**	**ACC TTC CTC CGG CAG CGG CA**
**tet_mini_F3**	**ATC TAA AGA GGA GAA AGG ATC TAT GCT GGG TAG CAC CTG GCT GAG CG**
**tet_mini_R3**	**CTG GAG ATC CTT ACT CGA GTT ATT AAC CTT CCT CCG GCA GCG GCA**
**Split PEs**	
**pBADuet_N-nCas9_F**	**GGA AGA TGC CAG GAA GAT AC**
**pBADuet_N-nCas9_R**	**CAC GGG TCA CAC ACC ATT ATC TTC G**
**pBADuet_C-nCas9_F**	**CAT TCA GAG AAG AAA CCA ATT G**
**pBADuet_C-nCas9_R**	**ACG GGT CAC ACA CCA TTA TC**
**pBADuet_Terminators_F**	**CGA AGA TAA TGG TGT GTG ACC CGT GTA CCC TTT ATG CGC TTC AG**
**pBADuet_Terminators_R**	**GGA CAA TTG GTT TCT TCT CTG AAT GTG AAA ACA ACG AGT GTC CTG**
**713N_F**	**TAA CGA AGA TAA TGG TGT GTG AC**
**713N_R**	**CAC TTG TGC TTT TTG AAT GTC**
**714C_F**	**TCT GGA CAA GGC GAT AGT TTA C**
**714C_R**	**CAT AGT GTA TTT CTC CTC TTT AAT CTC TAG**
**713N_gp41-1_F**	**AGA AGA CAT TCA AAA AGC ACA AGT GTG TCT GGA TCT GAA AAC CC**
**713N_gp41-1_R**	**CAC GGG TCA CAC ACC ATT ATC TTC GTT ATT CTT TAA CAT ACA GGC ACA TAC**
**713N_gp41-1+Junc_F**	**AGA AGA CAT TCA AAA AGC ACA AGT GTC AGG ATA CTG TCT GGA TC**
**gp41-1_714C_F**	**GAG ATT AAA GAG GAG AAA TAC ACT ATG CTG AAA AAA ATC CTG AAA ATC**
**gp41-1_714C_R**	**CAT GTA AAC TAT CGC CTT GTC CAG AAT TAT GGG TCA GAA TAT CAT TGG**

`

gp41-1+Junc_714C_R	CAT GTA AAC TAT CGC CTT GTC CAG AAC TGC TAG AAT TAT GGG TCA G
713N_NrdJ-1_F	AGA AGA CAT TCA AAA AGC ACA AGT GTG TCT GGT TGG TAG CAG CG
713N_NrdJ-1_R	CAC GGG TCA CAC ACC ATT ATC TTC GTT AAA TTG CAA CCA CCA GTT CAT CAT C
713N_NrdJ-1+Junc seq_F	AGA AGA CAT TCA AAA AGC ACA AGT GAA TCC GTG TTG TCT GGT TGG
NrdJ-1_714C_F	GAG ATT AAA GAG GAG AAA TAC ACT ATG GAA GCC AAA ACC TAT ATC
NrdJ-1_714C_R	CAT GTA AAC TAT CGC CTT GTC CAG AAT TAT GCA CCA GAA TAT CAT TG
NrdJ-1+Junc seq_714C_R	CAT GTA AAC TAT CGC CTT GTC CAG AGA TCT CTG AAT TAT GCA CCA G
573N_R	TTC TAT TTT TTT GAA ATA ATC TTC TTT TAA TTG C
574C_F	TGT TTT GAT AGT GTT GAA ATT TCA GG
674N_R	TTG CTT ATC CCT AAT ACC ATT AAT C
675C_F	TCT GGC AAA ACA ATA TTA GAT TTT TTG AAA TC
573N,674N_gp41-1_F	TGT CTG GAT CTG AAA ACC CAG
573N,674N_gp41-1+Junc_F	TCA GGA TAC TGT CTG GAT CTG
573N,674N_NrdJ-1_F	TGT CTG GTT GGT AGC AGC
573N, 674N_NrdJ-1+Junc_F	AAT CCG TGT TGT CTG GTT G
gp41-1_574C_R	CTG AAA TTT CAA CAC TAT CAA AAC AAC TAT TAT GGG TCA GAA TAT CAT TGG
gp41-1_574C+Junc_R	CTG AAA TTT CAA CAC TAT CAA AAC AAC TAC TGC TAG AAT TAT GGG TCA G
gp41-1_675C_R	AAA AAT CTA ATA TTG TTT TGC CAG AAT TAT GGG TCA GAA TAT CAT TG
gp41-1_675C+Junc_R	CAA AAA ATC TAA TAT TGT TTT GCC AGA ACT GCT AGA ATT ATG GGT CAG
NrdJ-1_574C_R	CTG AAA TTT CAA CAC TAT CAA AAC AAC TAT TAT GCA CCA GAA TAT CAT TGG
NrdJ-1_574C+Junc_R	CTG AAA TTT CAA CAC TAT CAA AAC AAC TGA TCT CTG AAT TAT GCA CCA G
NrdJ-1_675C_R	CAA AAA ATC TAA TAT TGT TTT GCC AGA ATT ATG CAC CAG AAT ATC ATT GG
NrdJ-1_675C+Junc_R	CAA AAA ATC TAA TAT TGT TTT GCC AGA GAT CTC TGA ATT ATG CAC CAG
573/574_P3-P4_F	AGA AGA TTA TTT CAA AAA AAT AGA AG
573/574_P3-P4_R	CTG AAA TTT CAA CAC TAT CA
674/675_P3-P4_F	GAT TAA TGG TAT TAG GGA TAA G
674/675_P3-P4_R	AAA AAT CTA ATA TTG TTT TGC C
713/714_P3-P4_F	AGA AGA CAT TCA AAA AGC AC
713/714_P3-P4_R	CAT GTA AAC TAT CGC CTT G
SpPE-pegRNAs	
pPEgRNA_F	GTT TAA ACG GTC TCC AGC

`

pPEgRNA_R	ACT AGT ATT ATA CCT AGG ACT G
lacZ_New_sub_F	GTC CTA GGT ATA ATA CTA GTT CAC CCG AGT GTG ATC ATC
lacZ_New_sub_R	AAG CTG GAG ACC GTT TAA ACA AAA AAA TTC TAG TTG GTT TAA CGC
lacZ_New_del_F	GTC CTA GGT ATA ATA CTA GTG AGC GAA CGC GTA ACG CG
lacZ_New_del_R	AAG CTG GAG ACC GTT TAA ACA AAA AAA TTC TAG TTG GTT TAA CGC GTA ACT AGA TAG
lacZ_New_ins_F	ATT CGC GTT ACG CGA CAA TAA GCG
lacZ_New_del_R	GTA AGT GCA GCG CGC ACC
pVRb_PEgRNA_backbone_F	TTT TTT TGA AGC TTG GGC CC
pVRb_PEgRNA_backbone_R	ACT AGT ATT ATA CCT AGG ACT GAG C
pPEgRNA_lacZ_GTtoTAsub_F	GTC CTA GGT ATA ATA CTA GTG CGA GTT GCG TGA CTA CCT AGT TTT AGA GCT AGA AAT AGC
pPEgRNA_lacZ_GTtoTAsub_R	GGG CCC AAG CTT CAA AAA AAA GTT GCG TGA CTA CTA ACG GGT AAC AGT GCA CCG ACT CGG TGC CAC TT
pPEgRNA_lacZ_CGdel_F	GTC CTA GGT ATA ATA CTA GTT ATG CAG CAA CGA GAC GTC AGT TTT AGA GCT AGA AAT AGC
pPEgRNA_lacZ_CGdel_R	GGG CCC AAG CTT CAA AAA AAG CAG CAA CGA GAC GTC AGA AAA TGC CGC TCA TGC ACC GAC TCG GTG CCA CTT
pPEgRNA_lacZ_TAGin_F	GTC CTA GGT ATA ATA CTA GTA ATC CCG AAT CTC TAT CGT GGT TTT AGA GCT AGA AAT AGC
pPEgRNA_lacZ_TAGin_R	GGG CCC AAG CTT CAA AAA AAC CGA ATC TCT ATC GTG CGT AGG TGG TTG AGC ACC GAC TCG GTG CCA CTT
ePEgRNA_F	GTT AAA CCA ACT AGA ATT TTT TTG AAG CTT GGG CC
pePEgRNA_GTtoTAsub_R	CGC GGG ATG ATA AGT TGC GTG ACT ACT AAC GG
GTtoTAsub_linker-motif	TAT CAT CCC GCG GTT CTA TCT AGT TAC GCG TTA AAC CAA CTA GAA
pePEgRNA_CGdel_R	GCG GGT GTA GGG CAG CAA CGA GAC GTC
CGdel_linker-motif	CCT ACA CCC GCG GTT CTA TCT AGT TAC GCG TTA AAC CAA CTA GAA
pePEgRNA_TAGin_R	CGC GTA TGT TGT CCG AAT CTC TAT CGT GCG TA
TAGin_linker_motif	ACA ACA TAC GCG GTT CTA TCT AGT TAC GCG TTA AAC CAA CTA GAA
tevopreQ1	AAG TGG CAC CGA GTC GGT GCC GCG GTT CTA TCT AGT TAC GCG TTA AAC CAA CTA GAA TTT T
tevopreQ1_F	CGC GGT TCT ATC TAG TTA CG
epegRNA_R	AAA AAA ATT CTA GTT GGT TTA ACG CGT AAC
IVT_T7	GAA ATT AAT ACG ACT CAC TAT AGG G
IVT_tevopreQ1	TTC TAG TTG GTT TAA CGC GTA AC

`

**pIVT_Insert_F**	**GAA ATT AAT ACG ACT CAC TAT AGG GGT TTC AGA GCT ATG CTG GAA ACA GC**
**pIVT_Insert_R**	**CGT AAC TAG ATA GAA CCG CGG CAC CGA CTC GGT GCC AC**
**pIVT_vector_R**	**CCC TAT AGT GAG TCG TAT TAA TTT CAG ATC TTT AGA ATT CCA GAA ATC ATC**
**T7 promoter_R**	**CCC TAT AGT GAG TCG TAT TAA TTT C**
**IVT_KanR_66-67_TGin_F**	**TAA TAC GAC TCA CTA TAG GGG GAG AGG CTA TTC GGC TAA CGT TTC AGA GCT ATG CTG GAA**
**IVT_KanR_66-67_TGin_R**	**CGT AAC TAG ATA GAA CCG CGC TAT TTC TGG CTA TTC GGC TAT GAC TGG GCA CAG CAC CGA CTC GGT GCC ACT T**
**IVT_KanR_281-282_AGdel_F**	**TAA TAC GAC TCA CTA TAG GGA AGG GAC TGG CTG CTA TAG TGT TTC AGA GCT ATG CTG GAA**
**IVT_KanR_281-282_AGdel_R**	**CGT AAC TAG ATA GAA CCG CGG ATC ATT CGA CTG GCT GCT ATT GGG CGA AGT GCC GCA CCG ACT CGG TGC CAC TT**
**pePEgRNA(opti)_KanR_66-67_TGin_F**	**GCT CAG TCC TAG GTA TAA TAC TAG TGG AGA GGC TAT TCG GCT AAC**
**pePEgRNA(opti)_KanR_281-282_AGdel_F**	**GCT CAG TCC TAG GTA TAA TAC TAG TAA GGG ACT GGC TGC TAT AGT**
**Construction of E. coli MG1655 mutants**	
**ybcC to pTarget_F oligo**	**GTC CTA GGT ATA ATA CTA GTA GGC CTT CAC GCT TCA TGC GGT TTT AGA GCT AGA AAT AGC**
**ybcC::KanR ID_F**	**TTC AGG CGA ATG CTG TTT AC**
**ybcC::KanR ID_R**	**TTG TCT GAG TTG ATA CTG GC**
**ybcC::KanR_up_F**	**CAC CTC TGA CTG AGA GTT TAC TCA C**
**ybcC::KanR_up_R**	**GAA GAT CCT TTT TGA TAA TCT GAA ATC CCG GGA GGT ACG AG**
**KanR::ybcC up_F**	**TCC CGG GAT TTC AGA TTA TCA AAA AGG ATC TTC ACC**
**KanR::ybcC down_R**	**CGT GAG GAT GCG GAA ACC ATT ATT ATC ATG ACA TTA ACC**
**ybcC::KanR_down_F**	**CAT GAT AAT AAT GGT TTC CGC ATC CTC ACG ATA ATA TCC G**
**ybcC::KanR_down_R**	**CAG CAG AGT AAA TCT TTG CCT CC**
**sbcB to pTarget_F oligo**	**GTC CTA GGT ATA ATA CTA GTT AAC TCG CGC TGG GAT TTA CGT TTT AGA GCT AGA AAT AGC**
**SaPE**	
**pCRISPR_SaPE2(Kim)_F1**	**GAT CTA AAG AGG AGA AAG GAT CTA TGA AGC GGA ACT ACA TCC TGG GC**
**pCRISPR_SaPE2(Kim)_F2**	**GCA CCC TCA GAT CAT CAA AAA GGG C**
**pCRISPR_SaPE2(Kim)_F3**	**AGC GGC GGC AGC AGC GGC GGC AGC AGC GGC AGC GAA ACC CCG GGC ACC AGC GAA AGC GCG**
**pCRISPR_SaPE2(Kim)_F4**	**GCA GCG GCG GTA GCA GCA CCC TGA ACA TCG AGG ACG AGT ATC**

`

**pCRISPR_SaPE2(Kim)_F5**	**GTG AAC GCT CTC TAC TAG AGT C**
**pCRISPR_SaPE2(Kim)_R1**	**GCC CTT TTT GAT GAT CTG AGG GTG C**
**pCRISPR_SaPE2(Kim)_R2**	**GCT GCT GCC GCC GCT GCC CTT TTT GAT GAT CTG AGG GTG C**
**pCRISPR_SaPE2(Kim)_R3**	**GCT GCT ACC GCC GCT GCT GCC GCC GCT GCT TTC CGG GGT CGC GCT TTC GCT GGT GC**
**pCRISPR_SaPE2(Kim)_R4**	**GAT ACT CGT CCT CGA TGT TCA GGG T**
**pCRISPR_SaPE2(Kim)_R5**	**CAT AGA TCC TTT CTC CTC TTT AGA TC**
**SaCas9_+151_A to C_F**	**CGC CCG CCG CCT GAA GCG CC**
**SaCas9_+151_A to C_R**	**CCG CGC TTG GAG CGG CGG CC**
**pCRISPR_SaPE2(STR)_F1**	**GAT CTA AAG AGG AGA AAG GAT CTA TGA AGC GCA ACT ACA TCC TG**
**pCRISPR_SaPE2(STR)_F2**	**AGC AGC GGC GGC AGC AGC GGC GGT AGC AGC ACC CTG AAC ATC GAG GAC GAG TAT C**
**pCRISPR_SaPE2(STR)_F3**	**GCA CCC GCA GAT CAT CAA GAA G**
**pCRISPR_SaPE2(STR)_R1**	**GCC CTT CTT GAT GAT CTG CG**
**pCRISPR_SaPE2(STR)_R2**	**GCT GCC GCT GCT GCC GCC GCT GCT GCC GCC GCT GCC CTT CTT GAT GAT CTG CGG GTG C**
**pCRISPR_SaPE2(STR)_R3**	**GAT ACT CGT CCT CGA TGT TCA GGG**
**SaCas9 (kim and STR) to pSaPE_F**	**ACG AAA ATT AGC TAG GGG GAA TAA TTA TGA AGC GGA ACT ACA TCC TG**
**clpB promoter_R**	**CAT AAT TAT TCC CCC TAG CTA ATT TTC**
**pSplitSA_F1**	**GTG GCA ATA GTC GTT TTG CAT G**
**pSplitSA_F2**	**CAG GAC ACT CGT TGT TTT CAC ATT CCT GAT ATT TTT GAC TAA ACC AAA TGC**
**pSplitSA_F3**	**ATG GAA GCC AAA ACC TAT ATC G**
**pSplitSA_F4**	**TAA AGA AGT CTC GTT CCG ACA GTT G**
**pSplitSA_R1**	**GAA TGT GAA AAC AAC GAG TGT CC**
**pSplitSA_R2**	**TGC CGA TAT AGG TTT TGG CTT CCA TGT TTA AAA CCT CCC TAT TTG ATG C**
**pSplitSA_R3**	**TGC CAA CTG TCG GAA CGA GAC TTC TTT AGT CAC CTC CTA GCT GAC**
**R.E)RTΔRH to pEPSA5_F**	**AAA TGA ATT CAT GAC CCT GAA CAT CGA GGA C**
**R.E)RTΔRH to pEPSA5_R**	**AAA ATC TAG ATT ACA GGC AGT TAT GCT GCA G**

`

PE_1_F	ATTAGCTAGGGGGAATAATTATGGATAAGAAATACTCAATAGGCTTAG
PE_1_R	GTCCAGACACTTGTGCTTTTTG
PE_2_F	CCATGATGATAGTTTGACATTTAAAGAAG
PE_2_R	AGTTTCCCCATTAGTTTCGATTAG
PE_3_F	ACTTGCAAATGGAGAGATTCG
PE_3_R	CTGTCGGAACGAGACTTCTTTACGGGCTGCTATTTTCAATC
PE_4_F	GATTGAAAATAGCAGCCCGTAAAGAAGTCTCGTTCCGACAG
PE_4_R	TCGATGTAACCCACTCGTG
PE_5_F	CTGGTGAAAGTAAAAGATGCTG
PE_5_R	CAGGAAAGAACATGTGAGCAAAAG
PE_6_F	CTTTTGCTGGCCTTTTGCTC
PE_6_R	CTATTGAGTATTTCTTATCCATAATTATTCCCCCTAGCTAATTTTC
PE_IDF_1	CTGATTCTGTGGATAACCGTATTACC
PE_IDF_2	GGAAGCGAGAAGAATCATAATG
PE_IDF_3	CGATTCTTTCTGCACGATTG
PE_IDF_4	GCATGCTATTTTGAGAAGACAAG
PE_IDF_5	CCATCAATCCATCACTGGTC
PE_IDF_6	GACGATACTCGTCCTCGATG
PE_IDF_7	TGCAGTACGTGGACGATCTG
PE_IDF_8	GTTGAAGCGCTGGTTAAGC
PE_IDF_9	GATAGACAGCACTGGCATGC
SaPE-pegRNAs	
Sa_gRNA_sacffold oligo	GTT TTA GTA CTC TGG AAA CAG AAT CTA CTA AAA CAA GGC AAA ATG CCG TGT TTA TCT CGT CAA CTT GTT GGC GAG A
pePEgRNA(Sa)_backbone_F	TTA TCT CGT CAA CTT GTT GGC GAG ACG CGG TTC TAT CTA GTT ACG
pePEgRNA(Sa)_backbone_R	GAT TCT GTT TCC AGA GTA CTA AAA CAC TAG TAT TAT ACC TAG GAC TGA GC
(Sa)_KanR_+66-67_TG ins_F	GTC CTA GGT ATA ATA CTA GTT TGT CTG TTG TGC CCA GTT AGG TTT TAG TAC TCT GGA AAC A
(Sa)_KanR_+66-67_TG ins_R	CGT AAC TAG ATA GAA CCG CGG GAG ATT ATG TTG TGC CCA GTC ATA GCC GAA TAG CCT CTC GCC AAC AAG TTG ACG A
(Sa)_lacZ_+766-767_CG to TA sub_F	GTC CTA GGT ATA ATA CTA GTC GGC GAG TTG CGT GAC TAC CTG TTT TAG TAC TCT GGA AAC A
(Sa)_lacZ_+766-767_CG to TA sub_R	CGT AAC TAG ATA GAA CCG CGG ATA ATA TGT TGC GTG ACT ACC TAT AGG TAA CAG TTC TCG CCA ACA AGT TGA CGA

`

**(Sa)_lacZ_+922-924_TAG ins_F**	**GTC CTA GGT ATA ATA CTA GTA AAC TGT GGA GCG CCG AAA TCG TTT TAG TAC TCT GGA AAC A**
**(Sa)_lacZ_+922-924_TAG ins_R**	**CGT AAC TAG ATA GAA CCG CGC TTA ATT TGG AGC GCC GAA ATC CCG TAG AAT CTC TAT CTC TCG CCA ACA AGT TGA CGA**
**(Sa)_lacZ_+1328 G del_F**	**GTC CTA GGT ATA ATA CTA GTG GCG ATG AGC GAA CGC GTA ACG TTT TAG TAC TCT GGA AAC A**
**(Sa)_lacZ_+1328 G del_R**	**CGT AAC TAG ATA GAA CCG CGT ATT ATT TAG CGA ACG CGT AAC GCA ATG GTG CTC TCG CCA ACA AGT TGA CGA**
**pePEgRNA(Sa)_mecA_F**	**GTC CTA GGT ATA ATA CTA GTT TAT TTT AAT AGT TGT AGT TGG TTT TAG TAC TCT GGA AAC A**
**pePEgRNA(Sa)_mecA_R**	**CGT AAC TAG ATA GAA CCG CGG ATA ATA TGT AGT TGT CGG GTT TGG TAT ATA TCT CGC CAA CAA GTT GAC GA**
**pePEgRNA(Sa)_mecA_R 2**	**CGT AAC TAG ATA GAA CCG CGG ATA ATA TGT AGT TGT CGG GTT TGG TAT ATA TTT TTA TGC TCT CGC CAA CAA GTT GAC GA**
**pePEgRNA(Sa)_mecA to pPEPSA5_F**	**AAC TTA CGA GTT ATA ATT AAA TCT TTT ATT TTA ATA GTT GTA GTT GGT TTT AG**
**pePEgRNA(Sa)_mecA to pPEPSA5_R**	**CAT CTA GTA CTC AAA TTA CAC TAT GAC AAC AGA TAA AAC GAA AGG**
**rrnB promoter_R**	**AAG ATT TAA TTA TAA CTC GTA AGT TTT TGT AAG**
**pDual_1_F**	**cgtaagtttttgtaagtcaagaattatttttaattattttGATGATAAGCTGTCAAACATGAG**
**pDual_1_R**	**CGTTTTCAGAGCAAGAGATTAC**
**pDual_2_F**	**GATCTTCTTGAGATCGTTTTGG**
**pDual_2_R**	**GAGAGTTAGGTTATTGGGATAAGTTAG**
**pDual_3_F**	**CAAAAATTGTATAAAGTGGCTCTAAC**
**pDual_3_R**	**CATAGTGTAATTTGAGTACTAGATGTTG**
**pDual_4_F**	**AGAATAATGTTGCTCATATCGTTTG**
**pDual_4_R**	**aaaataattaaaaataattcttgacttacaaaaacttacgagttataattaaatcttCAAAAAGAGTTTGTAGAAACGC**
**pDual IDF_F**	**GTCTGAGTTATAAAATAGATATCTCGG**
**pDual IDF_R**	**CAGACCAAGTTTACTCATATATACTTTAG**
**pDual_mecA_4_R**	**CCAACTAGAAtttttttCAAAAAGAGTTTGTAGAAACGC**
**pDual_mecA_F**	**GTTTCTACAAACTCTTTTTGaaaaaaaTTCTAGTTGGTTTAACGC**
**pDual_mecA_R**	**cgagttataattaaatcttATAGTTGTAGTTGTCGGGTAG**
**pDual_mecA_1_F**	**CTACCCGACAACTACAACTATaagatttaattataactcgtaagtttttgtaag**
**Construction of JE2 mutant**	
**mecA Up BamHI**	**aaaaggatccgcattttaatatcgattcaccataacg**
**mecA Up_R**	**cataaaaatatataccttacccgacaactacaac**

`

mecA Down_F	gttgtagttgtcgggtaaggtatatattttttatg
mecA Down XmaI	aaaacccgggctcttttgaactttagcatcaatagttag
pMAD IDF_F	GGAAGCGAGAAGAATCATAATG
pMAD IDF_R	GTTACGTTACACATTAACTAGACAG
mecA IDF_F	ctagtaaatattgtgctgccac
mecA IDF_R	gttactcatgccatacataaatgg
mecA IDF	gtgagcaatgaactgattatacttaac
NGS	
NGS)kanR_F	ACA CTC TTT CCC TAC ACG ACG CTC TTC CGA TCT CTT TCT TGC CGC CAA GGA TC
NGS)kanR_R	GTG ACT GGA GTT CAG ACG TGT GCT CTT CCG ATC TCG TCT TGC AGT TCA TTC AGG
NGS)mecA_F	ACA CTC TTT CCC TAC ACG ACG CTC TTC CGA TCT CAC AAA AAT TAT AAC ATT ATT TTG AC
NGS)mecA_R	GTG ACT GGA GTT CAG ACG TGT GCT CTT CCG ATC TTC GCT TTT AGA AAT ATA ACT G
In vitro Prime editing assay	
lacZ_mutant_F	GCT GGG GAA TTA ATC AGG C
lacZ_mutant_R	GAC CAG ATG ATC ACA CTC
check_F	TGA TTG AAG CAG AAG CCT GCG A
1393_edit_R	ATT AGC GCC GTG GCC TGA ATA
1393_W.T_R	ATT AGC GCC GTG GCC TGA ATC
